# Health system responsiveness to the mental health needs of Syrian refugees: mixed-methods rapid appraisals in eight host countries in Europe and the Middle East

**DOI:** 10.12688/openreseurope.15293.2

**Published:** 2024-05-30

**Authors:** Aniek Woodward, Daniela C. Fuhr, Alexandra S. Barry, Dina Balabanova, Egbert Sondorp, Marjolein A. Dieleman, Pierre Pratley, Samantha F. Schoenberger, Martin McKee, Zeynep Ilkkursun, Ceren Acarturk, Sebastian Burchert, Christine Knaevelsrud, Felicity L. Brown, Frederik Steen, Julia Spaaij, Naser Morina, Anne M. de Graaff, Marit Sijbrandij, Pim Cuijpers, Richard Bryant, Aemal Akhtar, Bayard Roberts, STRENGTHS consortium

**Affiliations:** 1 KIT Health, KIT Royal Tropical Institute, Amsterdam, 1092 AD, The Netherlands; 2 Athena Institute, Amsterdam Public Health Research Institute, Vrije Universiteit Amsterdam, Amsterdam, 1081 HV, The Netherlands; 3 Health Sciences, University of Bremen, Bremen, 28359, Germany; 4 Department of Health Services Research and Policy, Faculty of Public Health and Policy, London School of Hygiene and Tropical Medicine, London, WC1E 7HT, UK; 5 Department of Prevention and Evaluation, Leibniz Institute for Prevention Research and Epidemiology-BIPS, Bremen, 28359, Germany; 6 NHS England, London, SE1 8UG, UK; 7 Department of Psychology, Koc University, Sarıyer/İstanbul, Turkey; 8 Department of Education and Psychology, Division of Clinical Psychological Intervention, Freie Universität Berlin, Berlin, 14195, Germany; 9 Research and Development Department, War Child Holland, Amsterdam, 1098 LE, The Netherlands; 10 Amsterdam Institute of Social Science Research, University of Amsterdam, Amsterdam, 1018 WV, The Netherlands; 11 Department of Consultation-Liaison Psychiatry and Psychosomatic Medicine, University Hospital Zurich, University of Zurich, Zurich, 8091, Switzerland; 12 Department of Clinical, Neuro and Developmental Psychology, World Health Organization Collaborating Center for Research and Dissemination of Psychological Interventions, Amsterdam Public Health Research Institute, Vrije Universiteit Amsterdam, Amsterdam, 1081 HV, The Netherlands; 13 Babeș-Bolyai University, International Institute for Psychotherapy, Cluj-Napoca, Romania; 14 School of Psychology, University of New South Wales, Sydney, NSW 2052, Australia

**Keywords:** health system responsiveness, mental health, Syrian refugees, Europe, Middle East, comparative study, access, quality

## Abstract

**Background:**

Syrian refugees have a high burden of mental health symptoms and face challenges in accessing mental health and psychosocial support (MHPSS). This study assesses health system responsiveness (HSR) to the MHPSS needs of Syrian refugees, comparing countries in Europe and the Middle East to inform recommendations for strengthening MHPSS systems

**Methods:**

A mixed-methods rapid appraisal methodology guided by an adapted WHO Health System Framework was used to assess HSR in eight countries (Egypt, Germany, Jordan, Lebanon, Netherlands, Sweden, Switzerland, and Türkiye). Quantitative and qualitative analysis of primary and secondary data was used. Data collection and analysis were performed iteratively by multiple researchers. Country reports were used for comparative analysis and synthesis.

**Results:**

We found numerous constraints in HSR: i) Too few appropriate mental health providers and services; ii) Travel-related barriers impeding access to services, widening rural-urban inequalities in the distribution of mental health workers; iii) Cultural, language, and knowledge-related barriers to timely care likely caused by insufficient numbers of culturally sensitive providers, costs of professional interpreters, somatic presentations of distress by Syrian refugees, limited mental health awareness, and stigma associated to mental illness; iv) High out-of-pocket costs for psychological treatment and transportation to services reducing affordability, particularly in middle-income countries; v) Long waiting times for specialist mental health services; vi) Information gaps on the mental health needs of refugees and responsiveness of MHPSS systems in all countries. Six recommendations are provided to address these issues.

**Conclusions:**

All eight host countries struggle to provide responsive MHPSS to Syrian refugees. Strengthening the mental health workforce (in terms of quantity, quality, diversity, and distribution) is urgently needed to enable Syrian refugees to receive culturally appropriate and timely care and improve mental health outcomes. Increased financial investment in mental health and improved health information systems are crucial.

## Plain language summary

**Background:**

People who experience war often have increased mental health problems. Those who are forced to flee abroad frequently struggle to access adequate mental health and psychosocial support services. As a result, many refugees often do not seek or use such services.

Researchers of the Syrian REfuGees MeNTal HealTH Care Systems (STRENGTHS) consortium carried out rapid appraisals to assess the responsiveness of health systems to the mental health and psychosocial needs of Syrian refugees in eight countries: Egypt, Germany, Jordan, Lebanon, the Netherlands, Sweden, Switzerland, and Türkiye. They used quantitative and qualitative data, including primary and secondary data. This paper summarises and compares findings from the eight countries.

**What is health system responsiveness?:**

The ability of a health system to meet the expectations and needs of its people with regards to access, coverage, quality, and safety of services.

**What are our main findings and recommendations?:**

We found that all eight host countries struggle to provide responsive mental health and psychosocial support to Syrian refugees. We identified the following key challenges:

- Insufficient mental health providers and services, including uneven rural-urban distribution;
- Cultural, language, and knowledge-related barriers to timely care, caused by insufficient culturally sensitive providers and mental health stigma among Syrian refugee communities;
- Out-of-pocket costs for psychological treatment and transportation to services;
- Long waiting times for specialist mental health services;
- Information gaps on the mental health needs of Syrian refugees;

We recommend increasing national funding for mental health to help Syrian refugees to receive more culturally appropriate and timely care. Increased funding can reduce out-of-pocket payments by refugees, improve national health information systems, and strengthen the mental health workforce (in terms of quantity, quality, diversity, and distribution). We also recommend investment in cultural competency and mental health training for community-based workers and primary care providers.

## List of abbreviations

FGD: focus group discussion

HIC: high-income country

HSR: Health system responsiveness

MIC: middle-income country

mhGAP: Mental Health Gap Action Programme

MHPSS: mental health and psychosocial support

NGO: non-governmental organisation

PHC: primary healthcare

PTSD: post-traumatic stress disorder

RCT: randomised controlled trial

STRENGTHS: Syrian REfuGees MeNTal HealTH Care Systems

UNHCR: United Nations High Commissioner for Refugees

WHO: World Health Organization

## Introduction

The United Nations High Commissioner for Refugees (UNHCR) estimated that 108.4 million people worldwide were forcibly displaced end of 2022, including 35.5 million refugees and 5.4 million asylum seekers ^1^. Forcibly displaced populations bear a high burden of anxiety, depression, and post-traumatic stress disorder (PTSD) ^2– 5^. Refugees’ experiences of war and violence render them at risk of developing mental disorders, exacerbated by post-displacement stressors like poverty, inadequate accommodation, discrimination, social isolation, insecurity, and uncertainty about, for example, legal status, and potential for family reunification ^6– 8^.

Despite having high needs for mental health care, refugee populations have low utilisation of mental health and psychosocial support (MHPSS) ^9, 10^. MHPSS is defined as any type of external support that aims to protect or promote psychosocial well-being and/or prevent or treat mental disorders ^11^. It covers a broad range of services from diverse providers, including mental health specialists (e.g. psychiatrists, and psychotherapists) and non-specialists (e.g. community workers, social workers, and lay health workers). Previous research has found low uptake of MHPSS by refugees, who experience difficulties in accessing services because of stigma, unfamiliar language, low levels of trust, limited financial resources, and inadequate knowledge about where to seek support, as well as unavailability of relevant effective services ^9, 12– 15^. Their experiences point to major weaknesses in health system responsiveness (HSR) to the mental health needs of refugees.

There are several definitions of HSR, although there is a consensus that it involves not just the system’s capacity and ability to respond but also its actual response ^16^. The WHO conceptualisation of HSR is widely used ^16, 17^ and concerns the health system’s ability to meet the population’s legitimate expectations regarding non-medical aspects of their interactions with the system ^18^. These aspects include patient experiences with the choice of health providers, quality of amenities, and whether they are treated with dignity and confidentiality ^18^. Responsiveness is a complex construct and overlaps with elements of quality of care, coverage, access to care, equity in access, and patient satisfaction ^19, 20^. Individual expectations and experiences of the system shape perceived HSR, and consequently health behaviours and outcomes. Responsiveness is considered important for strengthening health system functioning, providing equitable and accountable services, and protecting the rights of citizens ^16^. A recent mapping found an increasing volume of research on HSR in the last decade, but also significant gaps in knowledge of how it is experienced by vulnerable groups (e.g. refugees) ^16^.

The Syrian REfuGees MeNTal HealTH Care Systems (STRENGTHS) study aims to strengthen mental health systems for Syrian refugees in Europe and the Middle East ^21^ and includes, among others, randomized controlled trials (RCT) of MHPSS interventions developed by the World Health Organization (WHO) and informed by consultations with affected populations. Syrians represent the largest refugee population globally, with 6.5 million hosted in 131 countries ^1^. The majority of Syrian refugees are based in neighbouring countries (i.e. Türkiye, Jordan, and Lebanon), but many have also sought refuge in high-income European countries, particularly Germany ^1^. The scope and protracted nature of the Syrian displacement has put pressure on the MHPSS systems of host countries, which can be expected to challenge responsiveness. The purpose of this paper is to assess HSR to the MHPSS needs of Syrian refugees, comparing countries in Europe and the Middle East to inform recommendations to strengthen MHPSS systems.

## Methods

### Study design and settings

We employed a rapid appraisal methodology to assess HSR to the MHPSS needs of Syrian refugees in all eight countries participating in the STRENGTHS study: Egypt, Germany, Jordan, Lebanon, the Netherlands, Sweden, Switzerland, and Türkiye. These countries collectively host 91% of the global Syrian refugee population (6.2 out of 6.8 million) ^22^.

Rapid appraisals are a pragmatic and systematic way to collect data across a range of health system domains in a feasible, timely and low-cost manner ^23, 24^. They are typically conducted by a small team (of two or more individuals) and triangulate data collected using a variety of methods and adopting different perspectives to identify key themes and areas of concern ^23, 25^. Rapid appraisals allow for selecting and combining primary and secondary data according to its relevance to the research objectives and context.

The multi-country rapid appraisal approach is appropriate for the STRENGTHS project, as an improved understanding of responsiveness of current health systems will inform decisions on ways to strengthen systems, for example, by integrating scalable psychological interventions ^21^. Multi-country studies give an insight into commonalities and differences across diverse contexts.

### Conceptual framework

**Figure 1.**
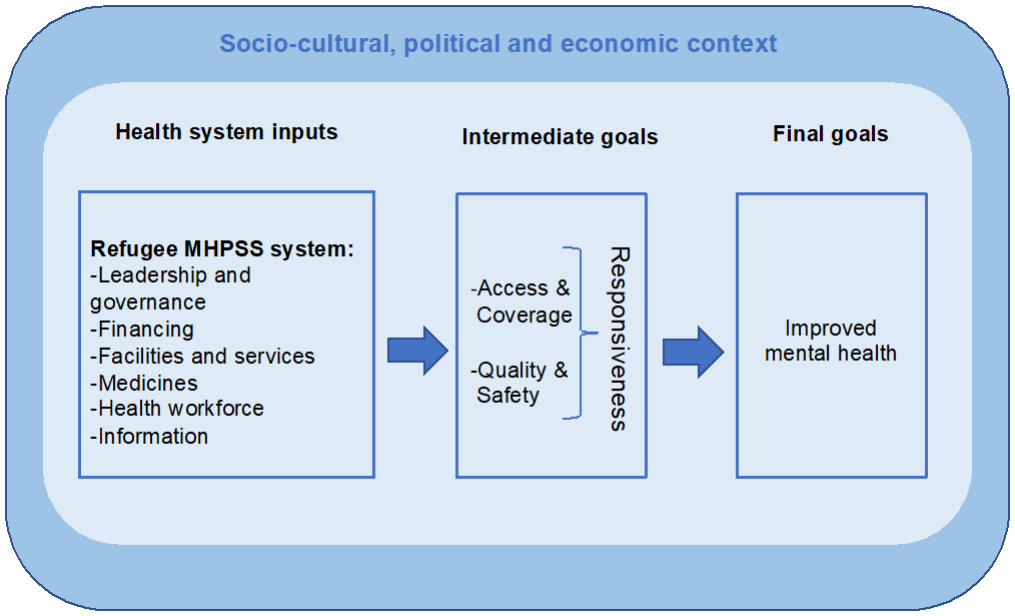
Conceptual framework on MHPSS system responsiveness to refugees’ needs, adapted from Fuhr, Roberts ^26^.

Our conceptual framework is set out in Figure 1. Details are published elsewhere ^26^ and can be found in Supplementary File 1 in the *Extended data* ^27^. In brief, it is based on WHO definitions of a health system, its building blocks, and HSR ^18, 28^ and domains identified in reviews on HSR ^17, 19^. While the WHO framework posits HSR as a final goal, in our framework HSR is an intermediate goal, with improved health outcomes positioned as final goals (see Figure 1). This is because the intermediate health goals defined by WHO (i.e. access, coverage, quality, and safety) interrelate with their eight domains of responsiveness (i.e. autonomy, choice, communication, confidentiality, dignity, quality of amenities, prompt attention, access to family and community support) ^26, 29^. Therefore we have used the intermediate goals as proxy measures for HSR. Definitions of the intermediate goals are found in Table 1.

**Table 1.** Definitions of intermediate health goals.

Goals	Definitions
**Access and** **coverage** **(seven** **elements)**	*i) Availability* – the volume (coverage) and type of existing services and whether this is adequate for the volume and needs of services users ^31^.
	*ii) Accessibility* – the relationship between the location of the services and the location of the people in need of them (e.g. transportation, travel time, distance, and cost) ^31^.
	*iii) Accommodation* – the relationship between the organisation of resources (appointment systems, hours of operation, walk-in facilities) and the ability of service users to accommodate to these factors ^31^.
	*iv) Affordability* – the prices of services in relation to the income of service users ^31^.
	*v) Acceptability* – the relationship of attitudes of service users about personal and practice characteristics of services to the actual characteristics of the existing services, including providers attitudes about acceptable personal characteristics of service users ^31^.
	*vi) Awareness* of mental health and information about available MHPSS services.
	*vii) Stigma* towards mental health and seeking MHPSS services, with stigma defined as the co-occurrence of labelling, stereotyping, separation, status loss, and discrimination in a context in which power is exercised ^32^.
**Quality**	The scope of care (and quantity) which is provided to the patient (i.e. the amount of care necessary to achieve a desired treatment outcome); the clinical quality of the service provided to the patients (e.g. cleanliness of the facility, skills and decision-making of the provider, equipment and supplies); service quality and acceptability of the service (e.g. convenience, interpersonal relationships) ^33^.
**Safety**	The degree to which healthcare processes avoid, prevent and ameliorate adverse outcomes or injuries that stem from the processes of healthcare itself ^34^.

The MHPSS system for refugees consists of two dominant subsystems: i) the ‘general MHPSS system’, which is commonly state governed and funded, and accessible to all citizens, including refugees with certain immigration statuses; and ii) the ‘parallel MHPSS system’, which is commonly run by non-governmental organisations (NGOs), civil society, and community groups and funded by UN organisations or from humanitarian budgets of donor countries. These parallel systems supplement care for vulnerable groups like refugees who are not (entirely) covered by or able to access the general MHPSS system. The type of MHPSS care provided in parallel systems is predominantly psychosocial support provided by non-specialists rather than specialist mental health services, which health professionals in the general system mainly provide. We recognise that in many contexts the MHPSS system is more complex. For example, in some countries there may be an overlap and linkages between these two subsystems, for example via referral pathways, while in other countries the private sector plays a large role in the health system ^30^. We applied the simplified framework to this comparative rapid appraisal.

### Data collection

In line with other rapid appraisals, a mixed-methods approach was used, with four different elements that used quantitative and qualitative methods, detailed below. Data collection and analysis was done iteratively by multiple team members, including local research partners. Supplementary File 2 in the *Extended data* gives an overview of all partner organisations and local ethical approvals ^27^.

i. Structured literature reviews

Literature reviews were conducted for all eight countries. Selected databases and grey literature sources were searched for relevant qualitative and quantitative studies published between Jan 2011 and Sept 2021. Eligibility criteria and search terms are presented in Supplementary File 3, *Extended data* ^27^. Database searches were conducted in English, with support from Endnote 20, and some grey literature searches in local languages (i.e. Dutch, German). Data from eligible studies was extracted and tabulated, listing source background (i.e. author, year, study country, objective, methods), summary of findings relevant to system responsiveness, and study population (i.e. refugees in general/asylum seekers/national population, country of origin, age). In total 436 studies were included in the literature reviews, covering single and multi-country studies. Most were conducted in Germany (n=135), followed by Jordan (n=94), Türkiye (n=52), the Netherlands (n=50), Sweden (n=50), Lebanon (n=49), Switzerland (n=27), and Egypt (n=13). A list of published sources, country level documents on specific indicators, and narrative reports on all countries are available on the STRENGTHS website. Supplementary File 4 in the *Extended data* contains all data extraction variables for the literature reviews ^27^.

ii. MHPSS system indicators

Additionally, we searched selected websites and databases to obtain indicators covering general and parallel MHPSS systems (see Supplementary File 3 in the *Extended data* for data sources ^27^). Indicators were derived based on the conceptual framework presented in Figure 1, and the Penchansky and Thomas ^31^ framework (Box 1). Supplementary File 5 in the *Extended data* shows the entire list of indicators ^27^. Relevant information was extracted and tabulated. If particular items of information could not be found, a search was conducted by local partners and, if not found, it was listed as ‘unknown’.

iii. Qualitative interviews

Qualitative interviews conducted in other components of the STRENGTHS project in the study countries were reanalysed for findings relevant to our study objectives. They included individual semi-structured interviews and focus group discussions (FGDs) with MHPSS providers, key-informants, and Syrian refugees. They had been conducted for three purposes: formative research for cultural adaptation of scalable psychological interventions; process evaluations of pilot and definitive RCTs of the psychological interventions; and scalability assessments of the psychological interventions. MHPSS providers and key informants were purposively sampled to include people from a variety of backgrounds and expertise. MHPSS providers included psychologists, psychiatrists, primary care providers, social workers and nurses working in mental health. Key informants included researchers, NGO workers, policy makers and advisors. Syrian refugees included a mix of those who had used MHPSS services before and those who had not. Syrian refugees interviewed were predominantly recruited from the STRENGTHS’ RCTs ( Table 2). In total 228 interviews with individuals were included in the analysis. Additionally, summaries of FGDs with Syrian refugees were included in Jordan (12 groups; n=72), the Netherlands (4 groups; n=14), and Switzerland (2 groups; n=20). All qualitative data was collected between May 2017 and October 2021.

**Table 2.** Individual interviews by type of respondent and country.

Country	Sample	MHPSS provider (PR)	Key informant (KI)	Syrian refugees (SR)	Total
Egypt		1	5	40	**46**
Germany		9	9	10	**27**
Jordan		14	7	15	**36**
Lebanon		3	0	0	**3**
Netherlands		36	17	22	**75**
Sweden		4	9	7	**13**
Switzerland		5	5	4	**14**
Türkiye		7	7	0	**14**
**Total**		**79**	**59**	**57**	**228**

iv. MHPSS access surveys

We also drew on data on previous utilisation of mental health care collected in the STRENGTHS project as part of the baseline for the RCT in Egypt (n=322), Germany (n=364), Lebanon (n=232), the Netherlands (n=206, Sweden (n=117), Switzerland (n=59), and Türkiye (n=368). Data for Jordan could not be used due to a data collection problem. In Egypt, Germany, the Netherlands, Sweden and Switzerland, the respondents were adult Syrian refugee women and men enrolled in the study with high levels of psychological distress (scoring above 15 on the 10-item Kessler Psychological Distress Scale (K10)) and functional impairment (scoring above 16 on the WHO Disability Assessment Schedule 2.0 (WHODAS 2.0)). For Lebanon, participants were official caregivers of children scoring 12 or above on the 17-item Paediatric Symptom Checklist. The respondents were asked whether they had experienced feelings such as anxiety, nervousness, depression, insomnia or any other emotional or behavioural problems since their displacement (and prior to their enrolment in the STRENGTHS RCT). If they had experienced these feelings, they were asked whether they had sought care for these feelings. Further details are available elsewhere ^35, 36^.

### Ethics approval

Ethical approval for primary data collection (i.e. qualitative interviews, MHPSS access surveys) was provided by the Ethics Committee of the London School of Hygiene & Tropical Medicine (14330 -1) in the UK. Additionally, local ethical approval was sought by STRENGTHS partners in all study countries and granted by local ethics boards (see Supplementary File 2 in the *Extended data* ^27^). All respondents in primary data collection gave written informed consent. Data protection, sharing, and confidentiality measures were in place. 

### Data analysis and synthesis

Primary and secondary data were extracted and analysed according to the MHPSS responsiveness conceptual framework ( Figure 1) ^26^. Quantitative primary data was analysed with Stata SE 17.0 and qualitative primary data was analysed with NVivo 12.6.1 and Atlas. TI 22.0.1. Since the interviews were collected primarily for other purposes, only units of data relevant to our conceptual framework were deductively coded and included in country reports. Preliminary findings were presented in country reports using a systematic narrative approach, structured according to our conceptual framework, and updated three times during the project (2017–2021). Several quality assurance processes were in place, such as training of interviewers (for primary data collection), verification of findings by local experts, and triangulation of methods. The final country reports were used to develop a comparative narrative synthesis.

The comparative synthesis drew on the approach for synthesis of multi-country qualitative research data used by Spicer, Aleshkina ^37^. This synthesis was led by KIT and LSHTM teams, and followed six phases: 1) Reading of country reports and developing rationale for focusing on selected categories for comparison by two analysists from KIT; 2) KIT and LSHTM teams agree on rationale, focus and approach; 3) Cross-country findings analysed and summarised by the first analyst (ASB) with support from the second analyst (AW). Summaries of key findings were tabulated; 4) The paper was drafted by the second analyst and circulated to KIT and LSHTM for feedback on clarity and coherence; 5) The revised paper was reviewed by country teams to check accuracy of study findings, add information where needed, and to agree on the synthesis.

## Results

Findings are structured according to the key elements in our conceptual framework, starting with the wider context, and ending with mental health outcomes. As we conceive HSR as overlapping with intermediate health goals, results are focused on these goals. Country reports with more detail on all elements of the framework, including used sources, are available on the STRENGTHS website.

### Socio-cultural, political, and economic context

Health systems are embedded in and influenced by the wider socio-cultural, political, and economic context. While data from all four methods were triangulated to develop synthesis findings on the context, most were drawn from structured literature reviews and MHPSS indicators. Supplementary File 6 in the *Extended data* contains more elaborate summary results on context and inputs, including references to original sources ^27^. Table 3 gives a summary overview of contextual indicators.

**Table 3.** Summary results on contextual factors.

	Egypt	Germany	Jordan	Lebanon	Sweden	Switzerland	The Netherlands	Türkiye
**GDP per capita in** **2020 (current USD)** **and income group**	3,569.2 USD Lower-MIC	46,252.7 USD HIC	4,282.8 USD Upper-MIC	4,649.5 USD Upper-MIC	52,274.4 USD HIC	87,100.4 USD HIC	52,396 USD HIC	8,536.4 USD Upper-MIC
**Total** **unemployment in** **2020 (% of total** **labour force)**	7.9%	3.8%	19.2%	11.4%	8.3%	4.8%	3.8%	13.1%
**Migration** **Integration Index in** **2019 (0-100) ^a^**	-	Health: 63 Overall: 58	-	-	Health: 83 Overall: 86	Health: 83 Overall: 50	Health: 65 Overall: 57	Health: 69 Overall: 43
**Languages spoken** **(%)**	Modern Standard (Egyptian) Arabic (68%), Sa’idi Arabic (29%). Arabic (2%)	German (94%), English (32%), French (9%); Russian (8%)	Arabic, English widely spoken	Lebanese Arabic (official), English and French widely spoken, Armenian	Swedish, and most able to speak English	(Swiss) German (63%), (Swiss) French (23%), (Swiss) Italian (8%), Romansh (<1%)	Dutch (98%), majority also speak English and German	Turkish (90%), Kurdish (6%), Arabic (1%), other (3%)
**The religion** **practised in country** **(%)**	Islam (95%), Christianity (5%), other (<1%)	Christianity (61%), non- religious (30%), Islam (4%), other (5%)	Islam (95%), Christianity (4%), other (1%)	Islam (54%), Christianity (40%), Druze (6%)	Christianity (63%), non- religious (35%), Islam (2%), other (<1%)	Christianity (68%), non-religious (24%), Islam (5%), other (3%)	non-religious (68%), Christianity (25%), Islam (5%), Hinduism and Buddhism (2%)	Islam (80%), Christianity (5%), non-religious (7%), other (8%)
**Number of** **registered Syrian** **refugees (year;** **% total host** **population)**	131,232 Syrian refugees registered (2021; 0.1%)	572,818 Syrian refugees registered (2019; 0.7%)	670,637 Syrian refugees registered (2021; 6.6%)	865,531 Syrian refugees registered (2020; 12.7%)	123,431 Syrian asylum seekers (2011–2020; 1.2%)	21,105 Syrian asylum seekers (2011–2020; 0.3%	27,284 Syrians granted residence permits (2010– 2020; 0.2%)	3,574,800 Syrian refugees registered (2021; 4.2%)
**Proportion of Syrian** **refugees living** **in camps in host** **country (%)**	Syrian refugees in Egypt do not live in camps but are living among Egyptian communities across Egypt.	Newly arrived Syrian refugees begin in a reception centre but eventually move into the community in one of the country’s sixteen states.	Approximately 17% of Syrian refugees live in camps.	Unknown as official camps (e.g. UNHCR camps) are not allowed.	Asylum seekers are offered accommodation by the Swedish Migration Agency (e.g. apartment, centre, house) or can live in private accommodation.	All asylum seekers live in one of the six federal reception and processing centres across the country for the first months before being settled into a host canton.	Asylum seekers live in one of the reception centres for the first months and are then housed by municipalities across the country.	Approximately 6% of Syrian refugees remain in refugee camps.

Sources: WHO, UNHCR, World Bank, World Atlas, Wikipedia (religions and languages), national statistical bureaus (e.g. CBS for the Netherlands, Migrationverket for Sweden). See Supplementary File 6 for a more detailed overview of findings on context and inputs, including sources used. ^a^MIPEX score is based on a set of indicators covering eight policy areas designed to benchmark current laws and policies against the highest standards through consultations with top scholars and institutions using and conducting comparative research in their area of expertise. The policy areas of integration covered by the MIPEX are labour market mobility; Family reunification, Education; Political participation; Permanent residence; Access to nationality; Anti-discrimination; and Health. A policy indicator is a question relating to a specific policy component of one of the eight policy areas. Each answer has a set of options with associated values (from 0 to 100). The maximum of 100 is awarded when policies meet the highest standards for equal treatment. Source: https://www.mipex.eu/

In brief our contextual analyses showed that the three upper middle-income countries (MICs) hosted the largest number of registered Syrian refugees, with the highest absolute number in Türkiye (3,574,800). In Lebanon, Syrian refugees made up 12.7% of the total population, followed by Jordan (6.1%) and Türkiye (4%). Many refugees are not registered so actual numbers may be much higher. Most Syrian refugees lived in host communities rather than camps, although in the four high-income countries (HICs) Syrian asylum seekers initially resided in reception centres or, in the case of Sweden, private accommodation or other accommodation provided by the Swedish Migration Agency. Egypt, Jordan, and Lebanon are, like Syria, predominantly Arabic-speaking and Muslim countries. Whether Syrian refugees can work depended on their legal status and country-specific regulations. Unemployment rates were much higher in host populations in MICs compared to HICs. Refugee children were entitled to education in all eight countries, however, many access barriers to education were reported (e.g. legal status, poverty, transportation, quality of education). Sweden had, according to the Migration Integration Policy Index, the most favourable political context (compared to the four HICs and Türkiye) in relation to the integration of refugees and their health.

### Health system inputs: refugee MHPSS system

Health systems can be represented as six building blocks (leadership and governance; financing; facilities and services; health workforce; medicines; and information), which through intermediate goals (access, coverage, quality, safety) reaches its final goal. Table 4 shows selected indicators on health system inputs.

**Table 4.** Summary results on health system inputs.

	Egypt	Germany	Jordan	Lebanon	Sweden	Switzerland	The Netherlands	Türkiye
**Leadership and** ** governance**								
*Mental health* * policy*	Yes	Yes	Yes	Unknown	Yes	No	Yes	Yes
*Mental health plan*	Yes	No	Yes	Unknown	Yes	Yes	Yes	Yes
*Mental health* * legislation*	Yes	No (covered in other laws)	No	Yes	Decentralised to municipalities	No (covered in other laws)	Yes	Yes
**Financing**								
*% GDP on health*	5.6%	11.4%	7.7%	6.0%	10.8%	11.9%	9.9%	4.1%
*% health* * expenditure on * *mental health*	0.5%	11.0%	Unknown	4.8%	Unknown	Unknown	10.7%	Unknown
*% mental health* * expenditure* * towards mental* * hospitals*	Unknown	11.3%	Unknown	54.0%	Unknown	Unknown	59.2%	Unknown
*% funding sources* * for health system*	Unknown	77% social security	8.8% social security, 30.7% out-of-pocket	52.5% social security, 36.4% out-of-pocket	Regional taxes, 13.8% out-of- pocket	71.5% social security, 26.8% out-of-pocket	93.0% social security, 10.1% out-of-pocket	Unknown
*Costs utilising* * MHPSS services for* * refugees*	None	None, but coverage varies	Same as uninsured Jordanians and coverage varies; no cost at UNHCR facilities	LBP 3,000 – 5,000 (1.75 – 3 EUR) for consultation at UNHCR facilities	Largely none, same cost as for Swedish citizens; can apply for reimbursement of out-of-pocket fees	None if approved by a doctor or psychiatrist	Same as Dutch citizens, pay the cost of monthly insurance premiums and out-of- pocket costs	Once registered, same as Türkiye’s citizens.
**Facilities and** ** services**	Ministry of Health main mental health service provider. MHPSS is not yet fully integrated into PHC, and so refugees either pay specialists or utilise NGO services in the parallel system.	All psychosocial services are available in the public sector. Alternatively, refugees have access to the parallel system in which many organisations offer counselling.	Syrian refugees can access MHPSS through primary care, which is increasingly integrated, or through UNHCR and NGO facilities that are accessible at no cost.	NGOs are extensively involved in care provision (in 2006, over 80% of the 110 PHC centres and 734 dispensaries are owned by NGOs). A variety of MHPSS is offered to the general population.	Refugees are identified and treated for mild mental health issues within PHC; Syrian refugees may be referred to providers who specialise in treating this group, who may be in a parallel system.	Syrian refugees with mental health needs are referred by nurses/social workers in asylum centres to PHC, where GPs can diagnose, treat, and refer. Referral is possible to general socio- psychiatric facilities, outpatient clinics for traumatised migrants, or tertiary care.	Early detection of mental health issues through prevention/ refugee integration programmes in the social domain; screenings at asylum centres; PHC providers in asylum centres or communities. GPs can treat or refer patients to secondary or tertiary care levels.	Syrians with temporary protection status can access PHC and community mental health centres for diagnosis, treatment, and referral. Those without temporary protection status can access emergency care in hospitals.
**Medicines**								
*Essential drugs list*	Yes	Yes	Yes	Yes	Yes, determined at the county level	Yes	No, but registered drugs list available and measures to keep them affordable.	Yes
*Psychotherapeutic * *medicines included* * in the list*	Yes	Yes, under ‘neurological diseases’	Yes	Yes	Dependent on county	Yes, under ‘neurological diseases’	N/A	N/A
*Cost of* * psychotherapeutic* * medicines*	Unknown; 80.0% of the population have free access	Covered by insurance	Unknown	Not covered by social insurance schemes, free at UNHCR facilities (but must pay consultation fee)	Up to €109 annually for drugs within the national insurance scheme, or €218 for drugs not covered	Unknown	Up to €385 annually	Unknown
*PHC doctors* * authorised* * to prescribe* * psychotherapeutic* * medicines*	Unknown	Yes	Unknown	Yes	Yes	Yes	Yes	Yes
**Health** ** workforce**								
*Mental health* * workers per* * 100,000*	8.40 mental health workers, 1.6 psychiatrists, 0.26 psychologists, 4.8 mental health nurses, 0.45 social workers	27.45 psychiatrists, 49.55 psychologists, 56.06 mental health nurses	7.8 mental health workers, 1.125 psychiatrists, 1.266 psychologists, 3.297 mental health nurses, 0.218 social workers	1.21 psychiatrists, 3.8 psychologists, 3.14 mental health nurses, 1.33 social workers	20.86 psychiatrists, 0.93 psychologists, 50.57 mental health nurses, 18.42 social workers	41.42 psychiatrists, 40.78 psychologists	24.23 psychiatrists, 90.76 psychologists, 2.87 mental health nurses	1.64 psychiatrists, 2.54 psychologists, 0.76 social workers
*Distribution of* * mental health* * professionals* * between urban* * and rural areas*	Uneven	Uneven	Relatively even	Unknown	Uneven	Unknown	Unknown	Unknown
*Mental health* * or cultural * *competency* * training*	Covers psychological first aid and basic mental health care	None for undergraduate; majority of PHC doctors have not received official in-service mental health training in last 5 years	Insufficient	3% of training for doctors, and 6% of training for nurses, is dedicated to mental health	Unknown, although nurses receive theoretical and clinical training in mental illness	Cultural vcompetency training is provided in under- and postgraduate curricula for medical students and residents	Care for patients with psychological complaints is part of GP training; nurses can specialise in mental health	Majority of PHC doctors have not received official in- service mental health training in last 5 years
**Information**								
*Data on the* * epidemiology of* * mental disorders* * collected at the* * national level*	Unknown	Yes	Unknown	Yes	Yes	No	Yes	Unknown
*Mental health data* * disaggregated * *refugees*	Unknown	No	Future plans to disaggregate health data by refugee status; Unknown if includes mental health	No	Unknown	No	No	Unknown
*NGO collection of* * refugee mental * *health data*	Unknown	Unknown	Unknown	Database of the Lebanon Crisis Response Plan encourages partners to collect disaggregated data for displaced Syrians, although includes limited data on mental health indicators	NGOs involved in MHPSS for refugees publish data within their annual reports	Unknown	NGOs involved in social support do not consistently publish data, aside from the Dutch Refugee Council. Knowledge centres Pharos and ARQ active in gathering and distributing information on refugee mental health	Unknown

Sources: WHO (especially Mental Health Atlas and WHO-AIMS reports), UNHCR, World Bank, Eurostat, reports by national governments. See Supplementary File 6 for more detailed overview of findings on context and inputs, including sources used.

In terms of information and governance, we found that information on the mental health of refugees was not routinely collected at national level in any country. All countries have a mental health policy or plan in place (unknown for Lebanon) and all, except Jordan, have specific mental health legislation. Mental health is integrated into primary health care (PHC) in the four HICs, and increasingly in the four MICs (e.g. mainly through expansion of the WHO Mental Health Gap Action Programme (mhGAP) involving training and supervision of non-specialist providers).

With regard to financing and services, we found that mental health received 0.5-11.0% (based on four countries) of national health budgets, which ranged from 4.1 to 11.9% of GDP across the eight countries. Health budgets were consistently lower in MICs compared to HICs. Important to note here is that MHPSS can be delivered outside of health facilities and is often not funded through health budgets. Syrian refugees who are granted asylum have the right to access similar MHPSS services under the same financial requirements as national citizens in Sweden, the Netherlands and Türkiye. In Germany and Switzerland local authorities determine financial access and coverage. In Jordan financial regulations for Syrian refugees for healthcare in governmental facilities changed regularly in recent years, with the latest regulation permitting refugees to access services for the same prices as uninsured Jordanians. Governmental PHC facilities and providers act as gatekeepers to specialist mental health services in the four HICs. In Türkiye, Lebanon, Jordan, and Egypt, PHC facilities are commonly run and financed or co-financed by NGOs, with MHPSS services increasingly integrated in their services. Refugees registered with the UNHCR or the government can access these NGO or government-NGO services generally at no cost and be referred to more advanced mental health care in the general health system. MHPSS services are more financially and legally restricted for unregistered refugees and asylum seekers compared to registered refugees and the host population. Separate facilities and services for children were not widely reported on, although in Lebanon it was noted that children with mental health needs are often first identified through awareness programmes in schools and referred through the same pathway as adults.

Assessment of the health workforce shows, as expected, that the four HICs had much higher numbers of psychiatrists and psychologists than the four MICs. The rural-urban distribution of the mental health workforce was rated in the WHO Assessment Instrument for Mental Health Systems as ‘disproportionate’ in Egypt and ‘relatively proportionate’ in Jordan. Other sources suggest this distribution was uneven in Germany and Sweden (for the other countries we rated this indicator as ‘unknown’). While NGOs serve as alternative health providers for Syrian refugees, particularly in the four MICs, there is limited publicly available data on the mental health workforce in parallel systems. Similarly, there are gaps in data on the mental health and cultural competency training of PHC nurses and doctors, although the limited information that we found may indicate training and supervision of non-specialists to be insufficient across all countries.

Finally in relation to medicines our appraisals confirmed that medicines for mental and behavioural disorders are available in all countries. Costs of medicines were either unknown or covered by health insurance up to a certain amount. PHC doctors in all countries were authorised to prescribe psychotherapeutic medicines (except for Jordan and Egypt where this was unknown).

### Intermediate goals: responsiveness

Access and coverage, and quality and safety are intermediate health system outcomes, affecting care seeking and eventually health outcomes. All four methods were used and combined to generate these findings. Findings drawn from secondary data (i.e. literature reviews and MHPSS indicators) are referenced with an original source. If no reference is provided, those findings are drawn from a primary data source (i.e. qualitative interviews and/or survey data from STRENGTHS study).

***Access & coverage.*** Availability and accessibility of mental health services were concerns in all countries. Demand outstrips supply of mental health resources, with insufficient numbers of mental health workers and/or services reported in Egypt, Jordan ^38– 40^, Türkiye, Switzerland ^41– 44^, Sweden ^45, 46^, Germany ^47– 50^, and in Lebanon:

*“I feel like there are many things missing [in MHPSS care]; there aren’t many available resources for them [Syrian refugees]…there aren’t enough staff in this domain.” (Health provider, Lebanon)*

Only the Netherlands reportedly had sufficient resources ^51, 52^, though it was noted that this was limited in rural areas ^53^. Egypt similarly reported rural areas having fewer providers and MHPSS services ^54^, while in Jordan there seemed to be a shortage in urban environments ^55^. The unequal distribution of resources contributes to accessibility barriers, and the need to travel to seek services was perceived as a barrier not only in the Netherlands but also in Sweden, Switzerland, Germany, Lebanon, Jordan, and Türkiye. Travel was described as a challenge due to the time it took, the distance required, safety, the cost of the journey, and cultural norms surrounding women traveling ^38, 56– 60^. Both Syrian men and women struggled to attend MHPSS services as explained by a key informant:

*“Women are going through a hard time. Some of them cannot leave their houses. Thus, they cannot attend activities. It’s hard reaching out to the male group because they are working.” (Key informant, Türkiye)*

Limited availability of culturally competent providers was reported in the Netherlands, Sweden, Germany, Switzerland, Egypt, and Türkiye. This is important, as acceptability seems to be a key issue across countries. Cultural and language barriers, as aspects of acceptability, were predominantly mentioned in European countries, likely due to the greater differences with Syrian society both culturally and linguistically ^47, 48, 53, 61– 66^. A Syrian refugee in the Netherlands highlighted this communication challenge:

*“They [primary health professionals] have been trying to find me a psychiatrist or psychologist to help me relieve the pains a little bit, but the problem is that the communication was very hard; that they couldn’t find me an Arabic[-speaking] doctor.” (Syrian refugee, the Netherlands)*

There was low recognition by medical professionals of mental health issues by Syrian refugees when these are presented primarily through somatic symptoms in Switzerland ^67^, the Netherlands ^53^, Sweden, and Germany. Language barriers were not an issue in Jordan, Egypt, and Lebanon, but despite many similarities, a failure to provide culturally sensitive services may still present a challenge.

Stigma and limited mental health awareness in the Syrian community were reported barriers across all countries in the published literature ^38, 41, 53, 60– 62, 68– 72^ and in our primary data. Our survey found that low mental health awareness, including beliefs about effectiveness of treatment and knowledge about where to seek help, generated barriers for Syrian refugees seeking support. It was widely reported that Syrian refugees may delay or refuse to seek support for mental health issues due to stigma rooted in a cultural belief that those who need MHPSS are “crazy” ^73^:

*“In my environment, I haven't ever heard of someone visiting a psychiatrist or even considering doing such no matter what they are going through [...] people may think you're crazy or something when you visit a psychiatrist” (Syrian refugee, Egypt)*

Recommendations to overcome cultural acceptability barriers include: expanding cultural sensitivity training for providers, which may decrease their use of stigmatising language ^74, 75^; increasing awareness around mental health in the Syrian community; and ensuring access to either a professional interpreter ^41– 44^ or Arabic-speaking doctor.

In terms of affordability, the costs associated with professional interpreters were perceived as a barrier by health providers in the Netherlands ^75^, Germany ^47, 48^, Türkiye, and Switzerland ^42^. In contrast in Sweden, all non-native speakers accessing the health system have the legal right to an interpreter hired by Swedish authorities. Additional affordability concerns for Syrian refugees accessing the health system in the Netherlands extend to paying costs in excess of those covered by health insurance, while Germany also imposes co-payments for treatment. Costs for specialist mental health services were only covered by health insurance for Syrian asylum seekers in Sweden if this was deemed ‘necessary’. Overall, affordability concerns seemed to be more prevalent in MICs, which could be contributed to their economic status and having less developed welfare systems. For example, caregivers of children in the STRENGTHS’ RCT in Lebanon reported perceived cost of services as the principal reason for not seeking care (79%). A MHPSS provider in Jordan commented about the unaffordability of private mental healthcare:

*“Are the mental health services actually available in Jordan? So we found that when you go to the mental health services that are available; they are only in private clinics with prices that may be too high.” (MHPSS provider, Jordan)*

In Egypt and Lebanon, Syrian refugees often have to pay out-of-pocket for psychological services in the general health system ^76^. In Jordan, out-of-pocket costs for public healthcare were reduced (subsidised 80%) due to a policy change in 2019, however, unaffordability of public health was reported to remain a significant barrier for Syrian refugees to accessing public health services ^77^. The cost of medications and transportation of people to health facilities in particular may be prohibitive in Jordan ^38, 56, 78^. Lebanon, Germany, Switzerland, the Netherlands, and Türkiye ^74^ also reported affordability barriers concerning the cost of transportation to services.

Information on accommodation was limited in the literature and therefore is solely based on primary data from the STRENGTHS RCTs. “Felt it would take up too much time/be inconvenient” was a principal reason for not seeking care reported amongst Syrian refugees participating in our MHPSS access surveys. Qualitative interview data further demonstrated that barriers to attending sessions of psychological interventions (in a research/RCT context) were lack of childcare and time, while facilitators were flexibility around appointment times, a convenient location, reimbursement of transportation costs, and the option of accessing the intervention online.

Information on access and coverage in parallel MHPSS systems was limited in all countries. While some psychosocial centres in study countries publish information on how many refugees they treat annually, they do not specify the proportions of people with a Syrian refugee background or provide information on access.

The findings from the MHPSS access survey data among adult Syrian refugee participants with elevated levels of psychological distress and functional impairment showed that 84% of them reported past emotional or behavioural problems since their displacement (ranging from 75% in Switzerland to 93% in Germany). Of these, 34% had previously sought care for these emotional problems (i.e., prior to enrolment in the STRENGTHS study) and this ranged from 24% in Egypt to 45% in Germany.

***Quality & safety.*** For Middle Eastern countries, limited information was available on quality and safety of MHPSS systems for refugees. Among European countries, the main issue is long waiting times. Long waiting times for mental health care were reported in the Netherlands ^53, 79^, Sweden ^80, 81^, Switzerland, Lebanon, Jordan ^71^, and Germany ^82^:

*“We don’t even have enough therapy places covered by health insurance anyway, so the waiting times are eternally long.” (Key informant, Germany)*

In the Netherlands, it was noted that efforts were being made to reduce waiting times in specialist mental healthcare ^83^. In Sweden, while 77% of patients accessed general health services within 90 days ^80, 81^, waiting lists for specialised psychiatric care were six to twelve months. This waiting time is similar to that reported for Germany ^82^.

In Switzerland, a major concern was timely diagnosis. This is in part due to the time it takes a Syrian refugee to seek help in the first place, and in part due to the long waiting times for services; however, it may also be due to somatic presentations of distress impacting initial diagnosis. As mentioned above, psychological concerns often present somatically among Syrian refugees ^67^, and if providers are not, or not sufficiently, trained in this they may miss cues and delay diagnosis and referral. Somatic presentations of distress amongst Syrian refugees were reported concerns in the Netherlands ^53^, Germany, and Sweden. While a subsequent treatment delay was not explicitly mentioned in these countries, some comments made by interviewees indicated this was a challenge:

*“When we [at NGO clinic] see [Syrian] patients, we often see that they have been circling around for quite some time in the health system. Because they have stomach problems, or headaches or pain in their body. And they have been sent around to various doctors to check the physical status. But it is rare for them to actually identify that there is something psychological behind the symptoms.” (Key informant, Sweden)*

Language barriers affected service quality in the Netherlands ^64, 65^, and consequently so did the financial barrier created by the use of professional interpreter services. The fact that interpreters are not covered by health insurance also affected quality of care in Germany ^47, 84, 85^. In Sweden, Syrian refugees were concerned that interpreters would not accurately communicate what the refugee wanted to express. In Switzerland, a lack of qualified interpreters was found to impact quality ^86, 87^:

*“And when the interpreters then tell me that the therapists themselves are overwhelmed and do not know and are somewhat chaotic and confused and talk for too long or too short, then I notice that often the interpreter setting does not work. That in reality it often is not ideally applied because multiple people have not been trained enough for this setting.” (Health provider, Switzerland)*

In Jordan ^88^ and Lebanon, the small mental health workforce compromised service quality. In Lebanon, there was a lack of choice and providers when seeking care. In Jordan, there was a particular lack of specialised and qualified mental health workers ^38, 57^, leading to limited screening and referral ^78, 89^. Both Lebanon and Jordan also lacked certain quality assurance mechanisms. For example, Lebanon had no certifying body for psychotherapy ^90^ while Jordan lacked a formal accreditation system ^78^. There was limited information available on quality and safety of MHPSS services for Syrian refugees in Egypt, although one Syrian refugee described the negative experience of being prescribed sleeping pills rather than receiving the psychological support they sought. This is supported by accounts from key informants of the system’s emphasis on psychiatric diagnosis and psychopharmacologic treatment rather than psychological support. There was no information available on quality and safety of MHPSS services for Syrian refugees in Türkiye.

Information on quality and safety in parallel MHPSS systems was limited in all countries. MHPSS services offered by NGOs and civil society organisations in Germany reportedly had long waiting lists. Similarly in Switzerland, long waiting times in the outpatient clinics for victims of war and torture were reported. Syrian refugees in Egypt commented positively on the quality of psychological services they received through the nongovernmental sector.

### Final goals: improve mental health

According to our conceptual framework, intermediate health outcomes influence health seeking behaviour and consequently the final goal, which are improved mental health outcomes. Studies included in our rapid appraisals typically did not make linkages between these outcomes or health system inputs. The majority of studies that reported on mental health outcomes focused on prevalence rates for depression, anxiety, and post-traumatic stress and were therefore used in this comparative synthesis. There were also studies reporting on other symptoms (e.g. prolonged grief disorder, psychological distress, psychological healthiness/wellbeing); however, these were excluded because they were less commonly reported and therefore more difficult to compare across countries.

We were not able to identify any nation-wide surveys of the prevalence of depression, anxiety, and post-traumatic stress among Syrian refugees in the study countries. Table 5 shows rates reported in several sub-national studies. Rates are for Syrian asylum seekers and refugees only. We excluded a considerable number of studies that did not present disaggregated data on Syrian refugees. Rates presented here need to be interpreted with caution: it is not an exhaustive list and rates are difficult to compare within and between countries due to differing methodologies, population groups studied (e.g. various age groups and stages of settlement), settings (e.g. camp-based, urban, health centre), and outcome measures (e.g. life-time or point prevalence; diagnostic criteria and tools). That said rates found amongst Syrian refugees and asylum seekers consistently appear much higher than those amongst the eight host populations, in which prevalence rates for depression ranges from 2.6 to 5.0% and for anxiety from 2.4 to 7.1% ^109– 111^ (no national figures available for post-traumatic stress). However, again caution is required in such comparisons given that the host population data are from Global Mental Health surveys, which have a different methodology.

**Table 5.** Observed prevalence (%) of common mental health symptoms for Syrian asylum seekers and refugees.

	Post- traumatic stress	Depression	Anxiety
Egypt	33.5 ^91^	30.0 ^91^	-
Germany	11.4 ^92^	14.5 ^92^	13.5 ^92^
Jordan	31.0-84.0 ^93– 98^	28.3-85.0 ^93, 99, 100^	50.0-84.0 ^93, 98, 99^
Lebanon	35.4-45.6 ^97, 101^	22.0 ^102^	-
The Netherlands	-	-	-
Sweden	29.9 ^103^	40.2 ^103^	31.8 ^103^
Switzerland	-	-	-
Türkiye	11.5-83.4 ^10, 104– 108^	12.5-70.5 ^10, 104– 108^	9.2-38.8 ^10, 107, 108^

Note: Observed prevalence as reported in selected studies identified in our structure review (published from 2015–2021). Please refer to individual papers for the different definitions and measures used for the mental health outcomes reported above.

## Discussion

The aim of this paper was to assess the responsiveness of health systems to the mental health needs of Syrian refugees in eight host countries. The prevalence of common mental disorders identified in our structured literature searches is in line with those found in systematic reviews and meta-analyses conducted amongst conflict-affected populations ^2, 112^, refugees and asylum seekers ^113, 114^, and Syrian refugees ^3, 15^. We found substantial heterogeneity, which can partly be explained by methodological differences. While there was no adequate information to link health system inputs with outcomes (intermediary and final), the poor outcomes found through our rapid appraisals are cause for concern and may point to health systems struggling to respond to the needs of Syrian refugees. Our study offers an insight into areas where responsiveness is particularly weak and how it may be improved.

Our conceptual framework explains that responsiveness to the mental health needs of individual Syrian refugees is achieved through intermediate health goals (access and coverage; quality and safety) and health system inputs (i.e. the six building blocks), which are embedded in a larger context (socio-cultural, political, economic). Therefore, a ‘response’ in our study means any action related to any of these levels (inputs, intermediate goals, context) that improves the mental health outcomes of Syrian refugees. Before we discuss ways to improve HSR, we first briefly discuss the main strengths and weaknesses in HSR towards the mental health needs of Syrian refugees.

### Strengths and weaknesses in HSR

Our analysis reveals two notable strengths in HSR towards mental health needs of Syrian refugees. The first is that mental health is integrated in PHC in HICs and increasingly integrated in MICs, which is in line with global recommendations ^115^. The second strength is that psychotherapeutic medicines were available in all study countries and, where known, PHC providers are able to prescribe them. These two strengths, however, have their limitations because of system-level resource and capacity challenges.

A shift from traditional hospital-based mental health care to primary care settings requires PHC providers (like GPs) to be competent in the detection, treatment, and referral of patients with mental health needs. Our findings indicate these competencies to be insufficient, because of limited mental health skills and cultural competency in PHC workers, limiting timely and appropriate diagnosis, treatment, and referral of Syrian patients. While in included HICs there were more mental health specialists, cultural and language barriers challenged the communication between Syrian refugees and health providers. This is mainly due to Arabic-speaking mental health professionals being limited in number and providing care through interpreters complicated (i.e. affordability, quality, confidentiality). Our findings showed that the availability of psychologists and psychotherapists was insufficient to meet the demand and therefore the health systems’ capacity for offering psychological therapies limited. In included MICs the number of mental health workers was even more limited and in addition systems in these countries may lack quality control structures for psychotherapy. Until these system-level resource and capacity challenges for psychotherapies are overcome, there is a danger that pharmacological treatment remains the dominant and only available treatment option for Syrian refugees with mental health needs in host populations.

Weaknesses in access and quality found in our study (e.g. stigma, language, cultural, knowledge/awareness, travel, costs, availability) are similar to those reported in reviews on access, barriers, and utilisation of MHPSS amongst refugees ^12, 13, 15^. While some weaknesses were more profound in certain countries, like cultural and language barriers in European countries, and out-of-pocket costs in MICs, most weaknesses were shared across contexts. This means our following recommendations for ways to increase HSR will be relevant to most, if not all, eight study countries as well as similar refugee contexts. The order in which the recommendations are listed do not reflect their order of priority.

### Recommendations

First, we recommend measures to strengthen the socio-economic situation of refugees. Our contextual analysis shows that employment of Syrian refugees was determined by existing policies; with rules varying by country, immigration status, work permits, and with fees for work permits possibly posing a financial obstacle for obtaining them. Similarly, our analysis highlighted that Syrian refugee children face access barriers to education. Unemployment, low income, and financial strain are social determinants of mental health and therefore can directly affect an individual’s mental health ^116^. Having policies, and particularly practices, in place that reduce access barriers to education and employment are essential to create viable livelihoods for Syrian refugees. This would not only benefit their mental health directly (i.e. reduced stress) and indirectly (i.e. financial capacity to pay for and travel to services), but also protect refugees from taking illegal jobs – further putting their health at risk – and positively affect the integration of refugees into host countries. Making such structural changes will be a challenging process requiring political will and collaboration between the health and other sectors, such as those involved with social security and education. However, subsequent to our rapid appraisal study some European countries such as the Netherlands have implemented less strict employment rules specifically for Ukrainian refugees who fled the 2022 war, which may be extended to other refugee populations.

Second, we recommend rapidly expanding the mental health workforce. This may be achieved through the further implementation of recommended approaches like mhGAP and collaborative task-sharing – involving the transfer of some mental health care responsibilities from specialists to non-specialists ^115, 117^. Task-sharing approaches have a potential to address shortages of mental health specialists and reduce waiting times for specialist mental health care, which were major quality concerns put forward by our cross-country examination. For example, in Türkiye mhGAP training of primary care doctors was found useful in responding to the mental health needs of Syrian refugees, although refresher trainings were recommended ^118^. Recent findings from the STRENGTHS project ^21^ have shown that task-sharing psychological interventions like Problem Management Plus can significantly minimise distress under Syrian refugee beneficiaries ^119^ and are scalable when taking into account key influencing factors and actors ^120– 123^. Similar results were found in a recent meta-analysis of the effectiveness of Problem Management Plus and its digital adaptation called Step-by-Step ^124^. That said, further research is needed on how to sustain the health benefits of such interventions ^125^.

Third, we recommend increasing the cultural competencies of the health workforce. The WHO recently developed Global Competency Standards to set a benchmark for the health workforce in providing culturally sensitive care to refugees and migrants ^126^. Cultural competence training of mental health specialists and non-specialists can help providers to recognise presentations of distress specific to Syrian culture and to better understand and respond to the fear and stigma associated with mental illness amongst Syrian communities ^14, 127, 128^. This again would enable more culturally appropriate and timely diagnosis, treatment, and referral. In non-Arabic speaking countries our results indicate it is vital in this regard to also increase the availability, affordability, and quality of professional interpreter services as well as to diversify the mental health workforce, such as through hiring Arabic-speaking health workers or cultural mediators. Participation of refugees themselves was reported important but underutilised in a review on current efforts to create more culturally sensitive refugee services ^129^, meaning this should be considered in future initiatives.

Fourth, we recommend increasing mental health awareness, including knowledge of mental health resources, and reducing mental health stigma among refugee communities. A recent Lancet commission calls for more action on ending stigma and discrimination in mental health worldwide and recommends the involvement of people with lived experience of mental health conditions in anti-stigma programmes ^130^. Other studies showed more research is needed on the effectiveness of anti-stigma interventions tailored to refugees ^128, 131^.

Fifth, we recommend strengthening national health information systems. Our results indicate that information on the mental health of Syrian refugees (and other refugees) was not routinely collected at national level in all study countries. Publicly available information on the parallel MHPSS systems was extremely limited. To offer a more holistic picture of the current situation, information from both general and parallel MHPSS systems need to be consolidated and interrelated by the national government and relevant UN agencies and NGOs. Besides data gaps, we found many data to be old and difficult to compare across countries. Reliable, timely, and disaggregated information is vital for health system actors (e.g. policymakers, managers service providers) to be more responsive to the needs and expectations of Syrian refugees (as well as refugees from other nationalities), as well as for measuring progress on health inequalities and for stimulating accountability mechanisms.

Sixth, and underpinning our other recommendations, we advise increasing national funding for mental health. Our appraisal showed that only 0.5–11.0% of national health budgets (which ranged from 4.1 to 11.9% of GDP) were allocated to mental health. This amount is disproportionate of the heavy individual, social, and economic burden of mental illness. Based on data from 28 EU countries, the OECD predicts the economic and social costs of mental ill-health to be more than 4% of GDP ^132^. As previously recommended ^115, 132^, more efficient use of mental health funding is needed, including more investment in community-oriented care ^133^. Strengthened MHPSS care in routine health and social care platforms has the potential to overcome some of the access barriers reported in this synthesis (e.g. unequal rural/urban distribution, physical access, and stigma).

### Limitations

Our study has several limitations. Firstly, publicly available data on some key indicators was scarce, with variations in data collection tools and years across countries challenging cross-country comparison and drawing definitive conclusions. Secondly, while reanalysis of primary qualitative data is pragmatic and time-reducing (and therefore befitting of rapid appraisals), it meant that not all interview data was relevant to our research objectives, nor did it help to fully address our objectives. Thirdly, our structured literature reviews included literature from various sources (e.g. peer-reviewed journals and grey literature like NGO reports) without performing quality assessments. While this led to inclusiveness and breadth in our synthesis, the results presented here may repeat those from poor-quality studies. A central issue in rapid appraisals is to find a “balance between speed and trustworthiness” ^25^. To account for these limitations, findings were discussed with country teams (who were involved in primary data collection and knowledgeable about the local context and literature) and the wider team of researchers involved in the synthesis. Also, triangulation of various data collection methods and perspectives validated our interpretations. Fourth, our study will be difficult to reproduce due to the number of methods and data used. To redress this, further information on our data can be found in the Supplementary Files, and authors can be contacted for more details. Fifth, since our focus was on MHPSS systems, issues such as social determinants of health were not addressed as much; however, we recognise their importance and recommend that this be a focus of future studies. Sixth, it was the beyond the scope of our analysis to compare HSR for Syrian refugees with citizens, however, this could be taken up by future research to generate further insights into common and refugee-specific bottlenecks in the MHPSS system.

## Conclusions

This study is the first to assess and compare HSR to the mental health needs of Syrian refugees across countries. Our rapid appraisals show that all eight host countries struggle to provide responsive MHPSS care to Syrian refugees. The many issues in access to quality care found in our synthesis may explain why Syrian refugees struggle to seek support and have poor mental health outcomes.

While various positive changes have been made in the general health systems of study countries, such as increasing the integration of mental health into primary care, many problems remain. Parallel nongovernmental MHPSS systems may be more responsive than state systems. However, in the long run, these parallel structures may be less sustainable and can undermine efforts to strengthen national health systems.

Strengthening the capacity of the mental health workforce (in quantity, quality, diversity, and distribution) is urgently needed to enable care-seeking for vulnerable populations like Syrian refugees and reduce waiting times in mental healthcare. Increased financial investment in mental health and improved health information systems (i.e. regularly updated disaggregated data) are crucial. More refugee-responsive MHPSS systems will benefit not just Syrian refugees and refugees of other nationalities but likely also migrants and host populations. A social determinants of health approach can be recommended to address the complex mental health needs of the refugees, and this may include MHPSS interventions as well as a range of socio-economic policies.

## Ethics and consent

Ethical approval for primary data collection (i.e. qualitative interviews, MHPSS access surveys) was provided by the Ethics Committee of the London School of Hygiene & Tropical Medicine (14330 -1) in the UK. Additionally, local ethical approval was sought by STRENGTHS partners in all study countries and granted by local ethics boards (see Supplementary File 2 in the *Extended data* ^27^). All respondents in primary data collection gave written informed consent. Data protection, sharing, and confidentiality measures were in place.

## Data Availability

Country reports with more detail on all elements of the framework, including used sources, are available on the STRENGTHS website. STRENGTHS research data are stored at VUA in a data repository. Access to primary data (anonymised qualitative data and survey data) is restricted for reasons of confidentiality. Access may be granted upon reasonable request to the STRENGTHS General Assembly ( e.m.sijbrandij@vu.nl). DataverseNL: Supplementary materials for "Health system responsiveness to the mental health needs of Syrian refugees: mixed-methods rapid appraisals in eight host countries in Europe and the Middle East”. https://doi.org/10.34894/DOMHPZ ^27^. This project contains the following extended data: Supplementary File 1 Detailed conceptual framework.docx (more detailed background information on our conceptual framework); Supplementary File 2 Overview partner organisations and ethics.docx (an overview of all organisations contributing to this study and local ethical approvals); Supplementary File 3 Eligibility criteria and search terms.docx (the eligibility criteria and search strategy used for structured literature searches); Supplementary File 4 Complete data extraction file.xlsx (the complete data extraction file of all included studies from literature searches); Supplementary File 5 Indicator checklist.docx (the checklist of specific indicators searched); Supplementary File 6 Detailed narrative findings on context and inputs.docx ( and more detailed narrative of findings on context and inputs, including references to sources). Data are available under the terms of the Creative Commons Attribution 4.0 International license (CC-BY 4.0).

## References

[1] UNHCR: Global Trends Report 2022. 2023. Reference Source

[2] Charlson F van Ommeren M Flaxman A : New WHO prevalence estimates of mental disorders in conflict settings: a systematic review and meta-analysis. *Lancet.* 2019;394(10194):240–8. 10.1016/S0140-6736(19)30934-1 31200992 PMC6657025

[3] Nguyen TP Guajardo MGU Sahle BW : Prevalence of common mental disorders in adult Syrian refugees resettled in high income Western countries: a systematic review and meta-analysis. *BMC Psychiatry.* 2022;22(1): 15. 10.1186/s12888-021-03664-7 34986827 PMC8729124

[4] Blackmore R Boyle JA Fazel M : The prevalence of mental illness in refugees and asylum seekers: a systematic review and meta-analysis. *PLoS Med.* 2020;17(9): e1003337. 10.1371/journal.pmed.1003337 32956381 PMC7505461

[5] Patanè M Ghane S Karyotaki E : Prevalence of mental disorders in refugees and asylum seekers: a systematic review and meta-analysis. *Glob Ment Health (Camb).* 2022;9:250–263. 10.1017/gmh.2022.29 36618716 PMC9806970

[6] Hynie M : The social determinants of refugee mental health in the post-migration context: a critical review. *Can J Psychiatry.* 2018;63(5):297–303. 10.1177/0706743717746666 29202665 PMC5912301

[7] Hajak VL Sardana S Verdeli H : A systematic review of factors affecting mental health and well-being of Asylum Seekers and Refugees in Germany. *Front Psychiatry.* 2021;12: 643704. 10.3389/fpsyt.2021.643704 33815176 PMC8012840

[8] Mesa-Vieira C Haas AD Buitrago-Garcia D : Mental health of migrants with pre-migration exposure to armed conflict: a systematic review and meta-analysis. *Lancet Public Health.* 2022;7(5):e469–e81. 10.1016/S2468-2667(22)00061-5 35487232

[9] Kaya E Karadag Caman O Kilic C : Need for and barriers to accessing mental health care among refugees in Turkey: a mixed methods study. *Eur J Public Health.* 2018;28(suppl_4). 10.1093/eurpub/cky213.453

[10] Fuhr DC Acarturk C McGrath M : Treatment gap and mental health service use among Syrian refugees in Sultanbeyli, Istanbul: a cross-sectional survey. *Epidemiol Psychiatr Sci.* 2019;29: e70. 10.1017/S2045796019000660 31727205 PMC8061186

[11] IASC: IASC guidelines on mental health and psychosocial support in emergency settings.Geneva: IASC;2007. Reference Source

[12] Satinsky E Fuhr DC Woodward A : Mental health care utilisation and access among refugees and asylum seekers in Europe: a systematic review. *Health Policy.* 2019;123(9):851–863. 10.1016/j.healthpol.2019.02.007 30850148

[13] Byrow Y Pajak R Specker P : Perceptions of mental health and perceived barriers to mental health help-seeking amongst refugees: a systematic review. *Clin Psychol Rev.* 2020;75: 101812. 10.1016/j.cpr.2019.101812 31901882

[14] Hassan G Ventevogel P Jefee-Bahloul H : Mental health and psychosocial wellbeing of Syrians affected by armed conflict. *Epidemiol Psychiatr Sci.* 2016;25(2):129–41. 10.1017/S2045796016000044 26829998 PMC6998596

[15] Hendrickx M Woodward A Fuhr DC : The burden of mental disorders and access to Mental Health and Psychosocial Support services in Syria and among Syrian refugees in neighboring countries: a systematic review. *J Public Health (Oxf).* 2020;42(3):e299–e310. 10.1093/pubmed/fdz097 31686110

[16] Khan G Kagwanja N Whyle E : Health system responsiveness: a systematic evidence mapping review of the global literature. *Int J Equity Health.* 2021;20(1): 112. 10.1186/s12939-021-01447-w 33933078 PMC8088654

[17] Mirzoev T Kane S : What is health systems responsiveness? review of existing knowledge and proposed conceptual framework. *BMJ Glob Health.* 2017;2(4): e000486. 10.1136/bmjgh-2017-000486 29225953 PMC5717934

[18] WHO: The world health report 2000. Health systems: improving performance.Geneva: WHO;2000. Reference Source 11910962

[19] Valentine NB de Silva A Kawabata K : Health system responsiveness: concepts, domains and operationalization.In: Murray CJ, Evans DV, editors. *Health Systems Performance Assessment: Debates, Methods and Empiricism.*Geneva: WHO;2003;573–93. Reference Source

[20] Valentine N Prasad A Rice N : Health systems responsiveness: a measure of the acceptability of health-care processes and systems from the user’s perspective.In: Smith P, Mossialos E, Papanicolas I, Leatherman S, editors. *Performance Measurement for Health System Improvement: Experiences, Challenges and Prospects.*London: WHO European Regional Office;2009;138–86. 10.1017/CBO9780511711800.007

[21] Sijbrandij M Acarturk C Bird M : Strengthening mental health care systems for Syrian refugees in Europe and the Middle East: integrating scalable psychological interventions in eight countries. *Eur J Psychotraumatol.* 2017;8(sup2): 1388102. 10.1080/20008198.2017.1388102 29163867 PMC5687806

[22] UNHCR: Refugee Data Finder 2022. Reference Source

[23] Beebe J : Basic concepts and techniques of rapid appraisal. *Human Organization.* 1995;54(1):42–51. 10.17730/humo.54.1.k84tv883mr2756l3

[24] Kumar K : An overview of rapid appraisal methods in development settings.In: Kumar K, editor. *Rapid appraisal methods.* Washington, DC: World Bank;1993. Reference Source

[25] McNall M Foster-Fishman PG : Methods of rapid evaluation, assessment, and appraisal. *Am J Eval.* 2007;28(2):151–68. 10.1177/1098214007300895

[26] Fuhr D Roberts B Woodward A : Health system responsiveness to the mental health needs of forcibly displaced persons.In: Bozorgmehr K, Roberts B, Razum O, Biddle L, editors. *Health Policy and Systems Responses to Forced Migration.* Switzerland: Springer International;2020;213–34. 10.1007/978-3-030-33812-1_12

[27] Woodward A Fuhr D Barry AS : Supplementary materials for "Health system responsiveness to the mental health needs of Syrian refugees: mixed-methods rapid appraisals in eight host countries in Europe and the Middle East.DataverseNL, V1. [Dataset].2022. 10.34894/DOMHPZ PMC11289593 39086733

[28] WHO: Everybody’s business. Strengthening health systems to improve health outcomes. WHO’s framework for action.Geneva: WHO;2007. Reference Source

[29] Papanicolas I Smith P : Health systems performance comparison. An agenda for policy, information and research.Maidenhead: University Press,2013. Reference Source

[30] Montagu D : The provision of private healthcare services in European countries: recent data and lessons for Universal Health Coverage in other settings. *Front Public Health.* 2021;9: 636750. 10.3389/fpubh.2021.636750 33791271 PMC8005513

[31] Penchansky R Thomas JW : The concept of access: definition and relationship to consumer satisfaction. *Med Care.* 1981;19(2):127–40. 10.1097/00005650-198102000-00001 7206846

[32] Hatzenbuehler ML Phelan JC Link BG : Stigma as a fundamental cause of population health inequalities. *Am J Public Health.* 2013;103(5):813–21. 10.2105/AJPH.2012.301069 23488505 PMC3682466

[33] Roberts MJ Hsiao W Berman P : Getting health reform right: a guide to improving performance and equity.Oxford: Oxford University Press;2008. 10.1093/acprof:oso/9780195371505.001.0001

[34] Kelley E Hurst J : Health care quality indicators project: conceptual framework paper.Paris: OECD;2006. Reference Source 10.1093/intqhc/mzl024 16954510

[35] de Graaff AM Cuijpers P Acarturk C : Effectiveness of a peer-refugee delivered psychological intervention to reduce psychological distress among adult Syrian refugees in the Netherlands: study protocol. *Eur J Psychotraumatol.* 2020;11(1): 1694347. 10.1080/20008198.2019.1694347 32082506 PMC7006761

[36] Uygun E Ilkkursun Z Sijbrandij M : Protocol for a randomized controlled trial: peer-to-peer Group Problem Management Plus (PM+) for adult Syrian refugees in Turkey. *Trials.* 2020;21(1): 283. 10.1186/s13063-020-4166-x 32192539 PMC7082999

[37] Spicer N Aleshkina J Biesma R : National and subnational HIV/AIDS coordination: are Global Health Initiatives closing the gap between intent and practice? *Global Health.* 2010;6: 3. 10.1186/1744-8603-6-3 20196845 PMC2838862

[38] IMC: Understanding the mental health and psychosocial needs, and service utilization of Syrian Refugees and Jordanian Nationals: a qualitative & quantitative analysis in the Kingdom of Jordan.Amman: International Medical Corps;2017. Reference Source

[39] Basheti IA Qunaibi EA Malas R : Psychological impact of life as refugees: a pilot study on a Syrian Camp in Jordan. *Trop J Pharm Res.* 2015;14(9):1695–701. 10.4314/tjpr.v14i9.22

[40] WHO, IMC: Assessment of mental health and psychosocial needs of displaced Syrians in Jordan.Eastern Mediterranean Public Health Network, Ministry of Health, World Health Organization, and International Medical Corps;2013. Reference Source

[41] Kiselev N Pfaltz M Haas F : Structural and socio-cultural barriers to accessing mental healthcare among Syrian refugees and asylum seekers in Switzerland. * Eur J Psychotraumatol.* 2020;11(1): 1717825. 10.1080/20008198.2020.1717825 32128044 PMC7034440

[42] Kiselev N Morina N Schick M : Barriers to access to outpatient mental health care for refugees and asylum seekers in Switzerland: the therapist's view. *BMC Psychiatry.* 2020;20(1): 378. 10.1186/s12888-020-02783-x 32680485 PMC7366894

[43] Müller F Roose Z Landis F : Psychische Gesundheit von Traumatisierten Asylsuchenden: Situationsanalyse und Empfehlungen. Bericht zuhanden des Bundesamtes für Gesundheit, Sektion Gesundheitliche Chancengleichheit.Luzern:: Interface;2018. Reference Source

[44] Kiselev N Pfaltz M Schick M : Problems faced by Syrian refugees and asylum seekers in Switzerland. *Swiss Med Wkly.* 2020;150: w20381. 10.4414/smw.2020.20381 33105021

[45] Lampa E Sarkadi A Warner G : Implementation and maintenance of a community-based intervention for refugee youth reporting symptoms of post-traumatic stress: lessons from successful sites. *Int J Environ Res Public Health.* 2020;18(1):43. 10.3390/ijerph18010043 33374648 PMC7793468

[46] Leiler A Wasteson E Holmberg J : A pilot study of a psychoeducational group intervention delivered at asylum accommodation centers-a mixed methods approach. *Int J Environ Res Public Health.* 2020;17(23): 8953. 10.3390/ijerph17238953 33271975 PMC7730684

[47] Bajbouj M Panneck P Winter SM : A Central Clearing Clinic to Provide mental health services for refugees in Germany. *Front Public Health.* 2021;9: 635474. 10.3389/fpubh.2021.635474 33634071 PMC7901997

[48] Böttche M Stammel N Knaevelsrud C : [Psychotherapeutic treatment of traumatized refugees in Germany]. *Nervenarzt.* 2016;87(11):1136–43. 10.1007/s00115-016-0214-x 27649983

[49] Biddle L Menold N Bentner M : Health monitoring among asylum seekers and refugees: a state-wide, cross-sectional, population-based study in Germany. *Emerg Themes Epidemiol.* 2019;16: 3. 10.1186/s12982-019-0085-2 31316579 PMC6613239

[50] Nikendei C Kindermann D Brandenburg-Ceynowa H : Asylum seekers' mental health and treatment utilization in a three months follow-up study after transfer from a state registration-and reception-center in Germany. *Health Policy.* 2019;123(9):864–72. 10.1016/j.healthpol.2019.07.008 31345581

[51] de Haan A Bloemen E Beekman J : Overzicht: preventieve interventies voor het versterken van de psychische gezondheid en veerkracht van statushouders. Wat kunt u inzetten in de gemeente? 2018. Reference Source

[52] Damen REC Dagevos J Huijnk W : Refugee reception re-examined: a quantitative study on the impact of the reception period for mental health and host country language proficiency among Syrian refugees in the Netherlands. *J Int Migr Integr.* 2022;23(1):1–21. 10.1007/s12134-021-00820-6 33814988 PMC8004562

[53] Beekman J : Dealing with the mental wellbeing of refugees in a Dutch context: a study about prevention and mental health care to refugees in the region Gelderland-Zuid.Nijmegen, the Netherlands: Radboud Universiteit;2017. Reference Source

[54] Egypt WaMoH: WHO-AIMS report on mental health system in Egypt.Cairo, Egypt;2006. Reference Source

[55] Akhtar A Engels MH Bawaneh A : Cultural adaptation of a low-intensity group psychological intervention for Syrian refugees. *Intervention, the Journal of Mental Health & Psychosocial Support in Conflict Affected Areas.* 2021;19(1):48–57. Reference Source

[56] Ansbro É Homan T Qasem J : MSF experiences of providing multidisciplinary primary level NCD care for Syrian refugees and the host population in Jordan: an implementation study guided by the RE-AIM framework. *BMC Health Serv Res.* 2021;21(1): 381. 10.1186/s12913-021-06333-3 33896418 PMC8074194

[57] Rizkalla N Arafa R Mallat NK : Women in refuge: Syrian women voicing health sequelae due to war traumatic experiences and displacement challenges. *J Psychosom Res.* 2020;129: 109909. 10.1016/j.jpsychores.2019.109909 31901581

[58] Karam E El Chammay R Richa S : Lebanon: mental health system reform and the Syrian crisis. *BJPsych Int.* 2016;13(4):87–9. 10.1192/s2056474000001409 29093915 PMC5619489

[59] Shedrawy J Lönnroth K Kulane A : ‘Valuable but incomplete!’ a qualitative study about migrants’ perspective on health examinations in Stockholm. *Int Health.* 2018;10(3):191–6. 10.1093/inthealth/ihy007 29474639

[60] Van Berkum M Smulders E Van den Muijsenbergh M : Zorg, ondersteuning en preventie voor nieuwkomende vluchtelingen: Wat is er nodig? [Care, support and prevention for newly arrived refugees: What is needed?].Utrecht: Pharos;2016. Reference Source

[61] Alawa J Zarei P Khoshnood K : Evaluating the provision of health services and barriers to treatment for chronic diseases among Syrian refugees in Turkey: a review of literature and stakeholder interviews. *Int J Environ Res Public Health.* 2019;16(15):2660. 10.3390/ijerph16152660 31349639 PMC6696441

[62] Doğan N Dikeç G Uygun E : Syrian refugees' experiences with mental health services in Turkey: "I felt lonely because I wasn't able to speak to anyone". *Perspect Psychiatr Care.* 2019;55(4):673–80. 10.1111/ppc.12400 31093988

[63] Mulders J Tuk B : Syrische nieuwkomers in de gemeente: Ervaringen van gezinnen met opvang, zorg en opvoeding [Newly arrived Syrians in the municipality: experiences of families with reception, care and education]Utrecht: Pharos;2016.

[64] Uiters E Wijga A : Gezondheid, leefstijl en zorggebruik [Health, lifestyle and care utilisation].In: Dagevos J, Huijnk W, Maliepaard M, Miltenburg E, editors *Syrie?rs in Nederland: Een studie over de eerste jaren van hun leven in Nederland [Syrians in the Netherlands: a study about the first years of their lives in the Netherlands]*. The Hague: Sociaal en Cultureel Planbureau;2018;174–92.

[65] Uiters E Wijga A : Syrische Statushouders op Weg naar Nederland - Gezondheid en zorggebruik [Syrian Statusholders on the Road to the Netherlands - Health and care utilisation]: Sociaal Cultureel Planbureau. 2021. Reference Source

[66] van Loenen T van den Muijsenbergh M Hofmeester M : Primary care for refugees and newly arrived migrants in Europe: a qualitative study on health needs, barriers and wishes. *Eur J Public Health.* 2018;28(1):82–7. 10.1093/eurpub/ckx210 29240907

[67] Morina N Kuenburg A Schnyder U : The association of post-traumatic and postmigration stress with pain and other somatic symptoms: an explorative analysis in traumatized refugees and asylum seekers. *Pain Med.* 2018;19(1):50–9. 10.1093/pm/pnx005 28340069

[68] Bartolomei J Baeriswyl-Cottin R Framorando D : What are the barriers to access to mental healthcare and the primary needs of asylum seekers? A survey of mental health caregivers and primary care workers. *BMC Psychiatry.* 2016;16(1):336. 10.1186/s12888-016-1048-6 27686067 PMC5041539

[69] Ekblad S : To increase Mental Health Literacy and human rights among new-coming, low-educated mothers with experience of war: a culturally, tailor-made group health promotion intervention with participatory methodology addressing indirectly the children. *Front Psychiatry.* 2020;11:611. 10.3389/fpsyt.2020.00611 32733291 PMC7362953

[70] Fleck F : Reforming mental health in Lebanon amid refugee crises. *Bull World Health Organ.* 2016;94(8):564–5. 10.2471/BLT.16.030816 27516633 PMC4969994

[71] Al-Rousan T Schwabkey Z Jirmanus L : Health needs and priorities of Syrian refugees in camps and urban settings in Jordan: perspectives of refugees and health care providers. *East Mediterr Health J.* 2018;24(3):243–53. 10.26719/2018.24.3.243 29908019

[72] Wells R Abo-Hilal M Steel Z : Community readiness in the Syrian refugee community in Jordan: a rapid ecological assessment tool to build psychosocial service capacity. *Am J Orthopsychiatry.* 2020;90(2):212–22. 10.1037/ort0000404 31414849

[73] Maconick L Ansbro É Ellithy S : "To die is better for me", social suffering among Syrian refugees at a noncommunicable disease clinic in Jordan: a qualitative study. *Confl Health.* 2020;14: 63. 10.1186/s13031-020-00309-6 32905304 PMC7465779

[74] Hassan G Kirmayer LJ Mekki-Berrada A : Culture, context and the mental health and psychosocial wellbeing of Syrians: a review for mental health and psychosocial support staff working with Syrians affected by armed conflict.Geneva: UNHCR;2015. Reference Source

[75] Zorginstituut Nederland: Tolkvoorziening voor anderstaligen in de geneeskundige geestelijke gezondheidszorg [Interpreter provision for other languages in medical mental healthcare]. 2020. Reference Source

[76] Lyles E Doocy S : Syrian refugee and affected host population health access survey in Lebanon.John Hopkins University and Medicines du Monde;2015.

[77] JRPSC: Jordan response plan for the Syria crisis 2020-2022.The Hashemite Kingdom of Jordan ministry of International Planning and Cooperation. Reference Source

[78] IMC and SIGI-JO: Mental Health and Psychosocial Support (MHPSS) needs assessment of displaced Syrians and host communities in Jordan international medical corps & sisterhood is global institute.2015.

[79] Vektis: Data bij factsheet GGZ wachttijden [data by factsheet mental healthcare waiting times]. 2021.

[80] Väntetider: Väntetider i vården [Waiting times in care]. 2021. Reference Source

[81] OECD: Waiting times for health services, next in line. 2020. Reference Source

[82] BPtK B : Ein Jahr nach der Reform der Psychotherapie-Richtlinie. 2018.

[83] GGZ Nederland: GGZ-partijen presenteren gezamenlijke aanpak om wachttijden in de geestelijke gezondheidszorg aan te pakken [Mental health stakeholders present shared approach to reduce waitting times in mental healthcare]. 2017.

[84] Asfaw BB Beiersmann C Keck V : Experiences of psychotherapists working with refugees in Germany: a qualitative study. *BMC Psychiatry.* 2020;20(1): 588. 10.1186/s12888-020-02996-0 33308187 PMC7733283

[85] Mewes R Kowarsch L Reinacher H : [Obstacles and opportunities for the psychotherapeutic treatment of asylum seekers]. *Psychother Psychosom Med Psychol.* 2016;66(9–10):361–8. 10.1055/s-0042-111314 27723926

[86] Bischoff A Hudelson P : Access to healthcare interpreter services: where are we and where do we need to go? *Int J Environ Res Public Health.* 2010;7(7):2838–44. 10.3390/ijerph7072838 20717543 PMC2922730

[87] Schoretsanitis G Eisenhardt S Ricklin ME : Psychiatric emergencies of asylum seekers; descriptive analysis and comparison with immigrants of warranted residence. * Int J Environ Res Public Health.* 2018;15(7):1300. 10.3390/ijerph15071300 29933607 PMC6068840

[88] IMC: Who is doing what, where and when (4ws) in mental health & psychosocial support in Jordan interventions mapping exercise October 2021.International Medical Corps;2021. Reference Source

[89] McNatt Z : “What’s happening in Syria even affects rocks”: a qualitative study of the Syrian refugee experience accessing noncommunicable disease services in Jordan. *Confl Health.* 2019; (13):26. 31210780 10.1186/s13031-019-0209-x PMC6567402

[90] El Chammay R Kheir W : Assessment of mental health and psychosocial support services for Syrian refugees in Lebanon. Beirut, Lebanon: UNHCR;2013. Reference Source

[91] Kira IA Shuwiekh H Rice K : A threatened identity: the mental health status of Syrian refugees in Egypt and its Etiology. *An International Journal of Theory and Research.* 2017;17(3):176–90. 10.1080/15283488.2017.1340163

[92] Georgiadou E Zbidat A Schmitt GM : Prevalence of mental distress among Syrian refugees with residence permission in Germany: a registry-based study. *Front Psychiatry.* 2018;9:393. 10.3389/fpsyt.2018.00393 30210373 PMC6121182

[93] Bryant R Giardineli L Bawaneh A : A lay provider delivered behavioral intervention for Syrian refugees and their children in Jordan. *Eur J Public Health.* 2020;30(Supplement_5). 10.1093/eurpub/ckaa165.626

[94] Beni Yonis O Khader Y Jarboua A : Post-traumatic stress disorder among Syrian adolescent refugees in Jordan. *J Public Health (Oxf).* 2020;42(2):319–24. 10.1093/pubmed/fdz026 30927431

[95] Sellouti M El Mehdi H Nguadi J : Risk factors for post-traumatic stress disorder among young Syrian refugee children in Jordan. *HSOA J Neonatol Clin Pediatr.* 2020. 10.24966/NCP-878X/100056

[96] Basheti IA Ayasrah SM Basheti MM : The Syrian refugee crisis in Jordan: a cross sectional pharmacist-led study assessing Post-Traumatic Stress Disorder. *Pharm Pract (Granada).* 2019;17(3):1475. 10.18549/PharmPract.2019.3.1475 31592018 PMC6763294

[97] Khamis V : Posttraumatic stress disorder and emotion dysregulation among Syrian refugee children and adolescents resettled in Lebanon and Jordan. *Child Abuse Negl.* 2019;89:29–39. 10.1016/j.chiabu.2018.12.013 30612072

[98] Halasa S Hamdan-Mansour AM Salami I : Post-traumatic stress and social anxiety among children of Syrian refugees in Jordan. *Int J Ment Health Addiction.* 2020;18:1611–9. 10.1007/s11469-020-00250-y

[99] Al-Fahoum A Diomidous M Mechill A : The provision of health services in Jordan to Syrian refugees. *Health Science Journal.* 2015;9(22):1–7. Reference Source

[100] Khader Y Bsbul M Assoboh L : Depression and anxiety and their associated factors among Jordanian adolescents and Syrian adolescent refugees. *J Psychosoc Nurs Ment Health Serv.* 2021;59(6):23–30. 10.3928/02793695-20210322-03 34060954

[101] Kazour F Zahreddine NR Maragel MG : Post-traumatic stress disorder in a sample of Syrian refugees in Lebanon. *Compr Psychiatry.* 2017;72:41–7. 10.1016/j.comppsych.2016.09.007 27732907

[102] Naal H Nabulsi D El Arnaout N : Prevalence of depression symptoms and associated sociodemographic and clinical correlates among Syrian refugees in Lebanon. *BMC Public Health.* 2021;21(1): 217. 10.1186/s12889-021-10266-1 33499834 PMC7836044

[103] Malm A : Post-migration stress and mental health among refugees : a population-based survey among refugees from Syria recently resettled in Sweden.Karolinska Institutet.;2021. Reference Source

[104] Acarturk C Cetinkaya M Senay I : Prevalence and predictors of posttraumatic stress and depression symptoms among Syrian refugees in a refugee camp. *J Nerv Ment Dis.* 2018;206(1):40–5. 10.1097/NMD.0000000000000693 28632513

[105] Al-Nuaimi S Aldandashi S Easa AKS : Psychiatric morbidity among physically injured Syrian refugees in Turkey. *Compr Psychiatry.* 2018;80:34–8. 10.1016/j.comppsych.2017.08.002 28972916

[106] Kaya E Kiliç C Karadağ Çaman Ö : Posttraumatic Stress and depression among Syrian refugees living in Turkey: findings from an urban sample. *J Nerv Ment Dis.* 2019;207(12):995–1000. 10.1097/NMD.0000000000001104 31658240

[107] Scherer N Hameed S Acarturk C : Prevalence of common mental disorders among Syrian refugee children and adolescents in Sultanbeyli district, Istanbul: results of a population-based survey. *Epidemiol Psychiatr Sci.* 2020;29: e192. 10.1017/S2045796020001079 33298230 PMC7737189

[108] Tekeli-Yesil S Isik E Unal Y : Determinants of mental disorders in Syrian refugees in Turkey versus internally displaced persons in Syria. *Am J Public Health.* 2018;108(7):938–45. 10.2105/AJPH.2018.304405 29771613 PMC5993388

[109] Institute for Health Metrics and Evaluation: Global Burden of Disease (GBD) results tool. 2019. Reference Source

[110] IHME: Global health data exchange. 2019. Reference Source

[111] WHO: Depression and other common mental disorders.Global Health Estimates; Geneva: WHO;2017. Reference Source

[112] Steel Z Chey T Silove D : Association of torture and other Potentially traumatic events with mental health outcomes among populations exposed to mass conflict and displacement: a systematic review and meta-analysis. *JAMA.* 2009;302(5):537–49. 10.1001/jama.2009.1132 19654388

[113] Henkelmann JR de Best S Deckers C : Anxiety, depression and post-traumatic stress disorder in refugees resettling in high-income countries: systematic review and meta-analysis. *BJPsych Open.* 2020;6(4):e68. 10.1192/bjo.2020.54 32611475 PMC7443922

[114] Turrini G Purgato M Ballette F : Common mental disorders in asylum seekers and refugees: umbrella review of prevalence and intervention studies. *Int J Ment Health Syst.* 2017;11(1): 51. 10.1186/s13033-017-0156-0 28855963 PMC5571637

[115] Patel V Saxena S Lund C : The *Lancet* commission on global mental health and sustainable development. *Lancet.* 2018;392(10157):1553–98. 10.1016/S0140-6736(18)31612-X 30314863

[116] Silva M Loureiro A Cardoso G : Social determinants of mental health: a review of the evidence. *Eur J Psychotriat.* 2016;30(4):259–92. Reference Source

[117] World Health Organization: mhGAP Intervention Guide Version 2.0. 2016. Reference Source

[118] Karaoğlan Kahiloğulları A Alatas E Ertuğrul F : Responding to mental health needs of Syrian refugees in Turkey: mhGAP training impact assessment. *Int J Ment Health Syst.* 2020;14(1): 84. 10.1186/s13033-020-00416-0 33292399 PMC7661162

[119] de Graaff AM Cuijpers P Twisk JWR : A peer-provided psychological intervention for Syrian refugees: results of a randomised controlled trial on the effectiveness of Problem Management Plus. *BMJ Ment Health.* 2023;26(1): e300637. 10.1136/bmjment-2022-300637 36789918 PMC10035776

[120] Woodward A Burchert S Barry AS : Scalability of digital psychological innovations for refugees: a comparative analysis in Egypt, Germany, and Sweden. *SSM Ment Health.* 2023;4: 100231. 10.1016/j.ssmmh.2023.100231

[121] Woodward A Sondorp E Barry AS : Scaling up task-sharing psychological interventions for refugees in Jordan: a qualitative study on the potential barriers and facilitators. *Health Policy Plan.* 2023;38(3):310–320. 10.1093/heapol/czad003 36631951 PMC10019561

[122] Woodward A de Graaff AM Dieleman MA : Scalability of a task-sharing psychological intervention for refugees: a qualitative study in the Netherlands. *SSM Ment Health.* 2022;2: 100171. 10.1016/j.ssmmh.2022.100171

[123] Spaaij J Fuhr DC Akhtar A : Scaling-up problem management plus for refugees in Switzerland - a qualitative study. *BMC Health Serv Res.* 2023;23(1): 488. 10.1186/s12913-023-09491-8 37189160 PMC10186708

[124] Schäfer SK Thomas LM Lindner S : World Health Organization's low-intensity psychosocial interventions: a systematic review and meta-analysis of the effects of problem management plus and step-by-step. *World Psychiatry.* 2023;22(3):449–462. 10.1002/wps.21129 37713578 PMC10503931

[125] Bryant RA Bawaneh A Awwad M : Twelve-month follow-up of a randomised clinical trial of a brief group psychological intervention for common mental disorders in Syrian refugees in Jordan. *Epidemiol Psychiatr Sci.* 2022;31: e81. 10.1017/S2045796022000658 36377410 PMC9677446

[126] WHO: Refugee and migrant health: global competency standards for health workers.Geneva: World Health Organization;2021. Reference Source

[127] McGrath M Acarturk C Roberts B : Somatic distress among Syrian refugees in Istanbul, Turkey: a cross-sectional study. *J Psychosom Res.* 2020;132: 109993. 10.1016/j.jpsychores.2020.109993 32172038

[128] Tahir R Due C Ward P : Understanding mental health from the perception of Middle Eastern refugee women: a critical systematic review. *SSM - Ment Health.* 2022;2: 100130. 10.1016/j.ssmmh.2022.100130

[129] Lau LS Rodgers G : Cultural competence in refugee service settings: a scoping review. *Health Equity.* 2021;5(1):124–34. 10.1089/heq.2020.0094 33778315 PMC7990563

[130] Thornicroft G Sunkel C Alikhon Aliev A : The *Lancet* commission on ending stigma and discrimination in mental health. *Lancet.* 2022;400(10361):1438–80. 10.1016/S0140-6736(22)01470-2 36223799

[131] Xin H : Addressing mental health stigmas among refugees: a narrative review from a socio-ecological perspective. *Univers J Public Health.* 2020;8(2):57–64. 10.13189/ujph.2020.080202

[132] OECD/European Union: Health at a glance: Europe 2018: state of health in the EU cycle.Paris/European Union, Brussels OECD Publishing;2018. 10.1787/health_glance_eur-2018-en

[133] WHO: World mental health report: transforming mental health for all.Geneva: World Health Organization;2022. Reference Source

