# Supplementary material for: Health system responsiveness to the mental health needs of Syrian refugees: mixed-methods rapid appraisals in eight host countries in Europe and the Middle East
Source: Open Res Eur. 2024 May 30;3:14. Originally published 2023 Jan 20. [Version 2] doi: 10.12688/openreseurope.15293.2 (PMC11289593; doi:10.12688/openreseurope.15293.2)
Supplement: Peer review history

# Reviewer report: Reviewer response for version 2

**DOI:** 10.21956/openreseurope.19158.r42054

**Published:** 2024-08-28

**Authors:** Asli Cennet Yalim

**Affiliations:** 1 School of Social Work, University of Central Florida, Orlando, Florida, USA

I would like to applaud the authors for their diligent effort and dedication to this study. The depth of research and attention to detail are evident throughout, and your work significantly contributes to the refugee mental health field. This manuscript demonstrates a strong commitment to advancing knowledge, and I look forward to seeing its continued impact.

 Here are some suggestions to improve the manuscript. To fully grasp the magnitude of the displaced Syrian population in these countries, it would be helpful to include estimated numbers of Syrians in each country.

- Regarding the study design, I believe the third paragraph should come before the second. First, the study design should be introduced, followed by a more detailed explanation of rapid appraisals. This is a strong approach, and I commend the researchers for their ambitious methodology. I am not sure if the conceptual framework has any short-term or immediate goals before the intermediate goals. If that’s the case, please justify why you only include intermediate goals in your framework. I think it would be helpful to list those indicators under the MHPSS system indicators section, rather than just referring to reference #27 and the figure.
- Please include the gender distribution in your text rather than only stating the number of adults. I believe the GDP per capita and total unemployment rates should be updated. The current data is from 2020 and is somewhat outdated. These percentages have changed significantly for some countries, such as Turkey's GDP.
- Please include a quote to support the following statements in your qualitative results: “There was low recognition by medical professionals of mental health issues by Syrian refugees when these are presented primarily through somatic symptoms in Switzerland67, the Netherlands53, Sweden, and Germany.
- Language barriers were not an issue in Jordan, Egypt, and Lebanon, but despite many similarities, a failure to provide culturally sensitive services may still present a challenge.” “In Jordan88 and Lebanon, the small mental health workforce compromised service quality. In Lebanon, there was a lack of choice and providers when seeking care. In Jordan, there was a particular lack of specialised and qualified mental health workers38,57, leading to limited screening and referral78,89. Both Lebanon and Jordan also lacked certain quality assurance mechanisms. For example, Lebanon had no certifying body for psychotherapy90 while Jordan lacked a formal accreditation system78. “  
- Under recommendations, I also recommend that the EU establish stronger oversight mechanisms for the countries receiving funding. How well do these countries achieve their MHPSS goals after receiving EU support for refugees? Is the reporting process transparent enough? The EU should develop a more structured control system to ensure these goals are met, especially in countries like Turkiye, Jordan, and Lebanon.

Is the study design appropriate and does the work have academic merit?

Yes

Is the work clearly and accurately presented and does it cite the current literature?

Yes

If applicable, is the statistical analysis and its interpretation appropriate?

Yes

Are all the source data underlying the results available to ensure full reproducibility?

Yes

Are the conclusions drawn adequately supported by the results?

Yes

Are sufficient details of methods and analysis provided to allow replication by others?

Yes

Reviewer Expertise:

Refugee mental health, Muslim refugees, access to mental health services

I confirm that I have read this submission and believe that I have an appropriate level of expertise to confirm that it is of an acceptable scientific standard, however I have significant reservations, as outlined above.

# Reviewer report: Reviewer response for version 2

**DOI:** 10.21956/openreseurope.19158.r42062

**Published:** 2024-07-30

**Authors:** Kriti Vashisht

**Affiliations:** 1 McNeese State University, Lake Charles, Louisiana, USA

The manuscript is prepared in a clear, coherent, and detailed manner. The introduction section is written well. The team connected the topics of refugees, mental health, MH programs, and health system responsiveness, in a comprehensive and brief way.

 Citations, methods, and data collection section are attempted well. I did notice a few missing citations like, "These parallel systems supplement care for vulnerable groups like refugees who are not (entirely) covered by or able to access the general MHPSS system."

 In the method section, it was helpful for the reader to read the definition of the rapid appraisal methodology. The data collection procedure is presented in a detailed way for example, how the data was collected; and  what data tools were used. I suggest writing the eligibility criteria for the literature review and MHPSS systems indicators in the manuscript even if the team has added the supplemental files. It will help in reading flow and understanding of the data without reviewing the extensive data sheets. 

 Further, I suggest that the team review the themes again, club the broad overlapping themes, and separate the broad themes, if one theme has multiple sub-themes. I recommend this article for indexing.

 Overall, it was a good read. I liked how the article was clear and easy to follow through. 

 Thank you for choosing me to review the article. Good luck to the authors!

Is the study design appropriate and does the work have academic merit?

Yes

Is the work clearly and accurately presented and does it cite the current literature?

Yes

If applicable, is the statistical analysis and its interpretation appropriate?

Yes

Are all the source data underlying the results available to ensure full reproducibility?

Yes

Are the conclusions drawn adequately supported by the results?

Yes

Are sufficient details of methods and analysis provided to allow replication by others?

Yes

Reviewer Expertise:

Human rights research; intersectionality research with health factors; participatory and qualitative methodologies; and the social and political practice of social work

I confirm that I have read this submission and believe that I have an appropriate level of expertise to confirm that it is of an acceptable scientific standard.

# Reviewer report: Reviewer response for version 2

**DOI:** 10.21956/openreseurope.19158.r42055

**Published:** 2024-07-30

**Authors:** Qusai Khraisha

**Affiliations:** 1 Trinity Centre for Global Health, Trinity College Dublin, Dublin, Ireland

The study by Woodward et al. provides a country-level assessment of health system responsiveness (HSR) to the mental health and psychosocial support (MHPSS) needs of Syrian refugees in eight host countries. The research employs a mixed-methods approach, combining qualitative and quantitative data to offer insights into the constraints faced by these health systems. However, there are several areas where the study could benefit from additional clarity, context-specific analysis, and refinement.

**Specific Comments**

1. **Geographical Scope (p. 1)**:
  - **Comment**: Egypt is not in the Middle East or Europe.
  - **Recommendation**: The authors should clarify the inclusion of Egypt in the study and adjust the geographical classification to accurately reflect the regions covered.
2. **Added Value and Novelty (p. 2)**:
  - **Comment**: The ‘constraints’ mentioned in the results found are very similar to previous research (discussed in the introduction), lacking clear added value.
  - **Recommendation**: Emphasise new findings or insights gained from this study that distinguish it from existing research. Addressing low trust in systems could be a novel addition.
3. **Sentence clarity (p. 2)**:
  - **Comment**: What constitutes a “long waiting time” is not defined.
  - **Comment**: The framing of somatic presentations of distress by Syrian refugees as an “issue” contributing to culture language and knowledge related barriers is not self-explanatory.
  - **Comment**: “widening rural-urban inequalities in the distribution of mental health workers” - It is also true that rural areas have less people so just because the number is unequal does not mean it is unfair.
  - **Recommendation**: Rewrite to improve clarity.
4. **Specificity on Syrians refugees (throughout)**:
  - **Comment**: Several time, the recommendations or discussions were applied to refugees in general.
  - “Information gaps on the mental health needs of refugees and responsiveness of MHPSS systems in all countries.” - information gaps of mental health needs of ALL refugees or just Syrians?
  - **Recommendation**: Given that this study on Syrian refugees, and that countries have different policies and MHPSS systems for different refugees, any vague sentences should be rewritten to focus on Syrian refugees specifically.
5. **HSR definition (p. 5)**:
  - **Comment**: “ability to meet the population’s legitimate expectations regarding non-medical aspects of their interactions with the system” - I do not follow, it says just before that the cocensus is not just about systems ability and then the definition given is about the ability.
  - **Recommendation**: Rewrite to improve clarity.
6. **Comparative Statements (p. 2)**:
  - **Comment**: The comparison in “All eight host countries struggle to provide responsive MHPSS” is unclear.
  - **Recommendation**: Specify the basis of comparison (e.g., struggle [relative to what] to the general public, to each other).
7. **Financial Investment (p. 2)**:
  - **Comment**: Recommendations for financial investment lack specificity and differentiation by country––it is unclear if Switzerland should have the same financial investment strategy as Egypt, for example.
  - **Recommendation**: Tailor recommendations for financial investment and health information systems to reflect the economic contexts of each country.
8. **Plain Language Summary (p. 2)**:
  - **Comment**: The plain language summary is not sufficiently simplified, it reads mostly identical to the abstract.
  - **Recommendation**: Simplify the summary further to ensure it is understandable to a general audience.
9. **Cultural Competency (p. 3)**:
  - **Comment**: The term “cultural competency” might need further explanation.
  - **Recommendation**: Define cultural competency and its relevance to MHPSS for Syrian refugees. 
10. **Justification for Country Inclusion (p. 5)**:
  - **Comment**: The rationale for including certain countries, like Switzerland, is unclear (all the other countries have among largest Syrian refugee populations, but not Switzerland… I expected Austria, for instance, if the justification is based on refugee numbers).
  - **Recommendation**: Provide a clear justification for the selection of each country included in the study.
11. **Conceptual Framework (p. 6)**:
  - **Comment**: The description of the MHPSS system for refugees may need citation.
  - **Recommendation**: Include citations for any general knowledge statements or clearly state if these are based on the study’s findings.
12. **Results Clarity and Analysis (p. 8)**:
  - **Comment**: At times, the results section is incohesive and difficult to follow. For example, it was confusing to read at the start of paragraph a country or a region and then to end the paragraph with a quote from another country or region. It was also unclear why in some paragraphs, only specific countries were mentioned and not others. It was unclear why some surprising results were not discussed (limited cultural competency in Egypt), a country sharing similar religion and language. Vague terms like ‘largely,’ and ‘seem’ were frequently used.  Some ideas in paragraphs appeared underdeveloped (some were only 1 sentence, and many were 2 sentences).
  - **Recommendation**: Improve the structure by developing ideas more thoroughly and grouping related findings into coherent paragraphs.
  - **Comment**: With the exception of the “Intermediate goals: responsiveness” section, the results generally were not broken down by country, leading to a lack of specificity.
  - **Recommendation**: Structure the results section to compare countries not just with similar issues but highlight how countries differ. Provide analyses that goes beyond mere the GDP of a country (HIC versus MIC or LIC).
13. **Contextual Analysis (p. 8)**:
  - **Comment**: The analysis of the socio-cultural context is superficial.
  - **Recommendation**: Provide a more detailed examination of how socio-cultural, political, and economic contexts differ across countries and impact MHPSS.
14. **Financial Data Variation (p. 10)**:
  - **Comment**: The variation in health budgets is significant across countries but the result section lumps all countries together without any further elaboration or deep analysis (e.g., that “health budgets were consistently lower in MICs compared to HICs” is not a deep analysis).
  - **Recommendation**: Discuss the implications of this variation and how it affects MHPSS delivery in different countries.
15. **Refugee Definition (p. 10)**:
  - **Comment**: The term “refugee” needs clarification.
  - **Recommendation**: Clearly define how refugees were identified and included in the study as opposed to resettled Syrians. Was the 1951 definition applied?
16. **Comparative Analysis (p. 10)**:
  - **Comment**: The intermediate goals section is the best analysis section.
  - **Recommendation**: Use this section as a model for improving the depth and clarity of other sections.
17. **Discussion and Specific Recommendations (p. 16-17)**:
  - **Comment**: Based on the added-value of this study, being the first to be conducted several across countries, it was surprising that the discussion lacks comparability of context. Similarly, recommendations were not country-specific or that they did not integrate country context.
  - **Recommendation**: Include more comparative analysis of contextual differences and their impact on MHPSS and tailor recommendations to the specific contexts and capacities of each country.
18. **Evidence for Claims (p. 18)**:
  - **Comment**: The claim that more refugee-responsive MHPSS systems will benefit other groups needs substantiation.
  - **Recommendation**: Provide evidence or references to support this claim.
19. **Conclusion on Social Determinants (p. 18)**:
  - **Comment**: The recommendation to address social determinants of health seems inconsistent with the limitations stated: “as social determinants of health were not addressed as much” not consistent with “a social determinants of health approach can be recommended to address the complex mental health needs of the refugees, and this may include MHPSS interventions as well as a range of socio-economic policies.”
  - **Recommendation**: Align the conclusion with the study’s findings and limitations or provide a clear rationale for the recommendation.

**Conclusion**

 The study by Woodward et al. is a valuable contribution to the field of refugee mental health. Addressing the noted weaknesses and incorporating the suggested improvements could further enhance the impact and applicability of the research. By refining the structure, providing clearer definitions and justifications, and tailoring recommendations to specific country contexts, the study can offer more precise and actionable insights for policymakers and practitioners.

Is the study design appropriate and does the work have academic merit?

Yes

Is the work clearly and accurately presented and does it cite the current literature?

Partly

If applicable, is the statistical analysis and its interpretation appropriate?

Not applicable

Are all the source data underlying the results available to ensure full reproducibility?

Yes

Are the conclusions drawn adequately supported by the results?

Partly

Are sufficient details of methods and analysis provided to allow replication by others?

Partly

Reviewer Expertise:

I research refugee mental health and parenting behaviours. I have also worked at UNHCR.

I confirm that I have read this submission and believe that I have an appropriate level of expertise to confirm that it is of an acceptable scientific standard, however I have significant reservations, as outlined above.

# Reviewer report: Reviewer response for version 2

**DOI:** 10.21956/openreseurope.19158.r41077

**Published:** 2024-06-14

**Authors:** Mathilde Mathilde Sengoelge

**Affiliations:** 1 Department of Global Public Health,, Karolinska Institutet, Stockholm, Sweden

I agree it is now ready for indexing.

Is the study design appropriate and does the work have academic merit?

Partly

Is the work clearly and accurately presented and does it cite the current literature?

Yes

If applicable, is the statistical analysis and its interpretation appropriate?

Not applicable

Are all the source data underlying the results available to ensure full reproducibility?

Yes

Are the conclusions drawn adequately supported by the results?

Yes

Are sufficient details of methods and analysis provided to allow replication by others?

Partly

Reviewer Expertise:

Mental health of refugees resettled in high income countries.

I confirm that I have read this submission and believe that I have an appropriate level of expertise to confirm that it is of an acceptable scientific standard.

# Reviewer report: Reviewer response for version 1

**DOI:** 10.21956/openreseurope.16535.r35136

**Published:** 2023-09-18

**Authors:** Mathilde Mathilde Sengoelge

**Affiliations:** 1 Department of Global Public Health,, Karolinska Institutet, Stockholm, Sweden

Thank you for the opportunity to review this article with key recommendations on how to improve the HSR of Syrian refugees in order to strengthen their mental health.

 Suggestions for improvement:

 Abstract: highlight that the article provides 6 recommendations as results is dominated by the constraints/problems which are known to many in the field.

 Manuscript:

 Conceptual Framework: The WHO framework published in 2007 which you cite has 'improved health' as an overall goal, alongside three other important ones. The Figure 1 in this article does not make it clear what is the difference between the 'general' and 'parallel' inputs without reading the text, yet it should be understood as stand alone and informative. As a reviewer I was unable to access reference 26 through my university library in order to understand why it is necessary to detail all 6 for all citizens vs. for refugees only. The suppl. material provided do not help to clarify this point. As such, Framework 1 inputs would benefit from editing. There is furthermore no link between this framework and the presentation of the results, health system inputs as these were not structured as 'general' and 'parallel'.

Is the study design appropriate and does the work have academic merit?

Partly

Is the work clearly and accurately presented and does it cite the current literature?

Yes

If applicable, is the statistical analysis and its interpretation appropriate?

Not applicable

Are all the source data underlying the results available to ensure full reproducibility?

Yes

Are the conclusions drawn adequately supported by the results?

Yes

Are sufficient details of methods and analysis provided to allow replication by others?

Partly

Reviewer Expertise:

Mental health of refugees resettled in high income countries.

I confirm that I have read this submission and believe that I have an appropriate level of expertise to confirm that it is of an acceptable scientific standard, however I have significant reservations, as outlined above.

# Response

**Published:** 2024-05-01

**Authors:** Aniek Woodward

**Affiliations:** KIT Royal Tropical Institute, The Netherlands

Dear Ms. Sengoelge,   Thank you for taking the time to review our paper. In response to your valuable feedback we have made the following changes to our paper:

- Figure 1 has been adapted so that the inputs make clear they cover the “refugee MHPSS system” (see Figure at start of “conceptual framework” under our Methods section). According to our conceptualisation the refugee MHPSS covers two dominant subsystems: a general system and a parallel system. Together these describe the ways refugees can typically access health systems in host countries (i.e. the refugee MHPSS system). Our results are focused on the refugee MHPSS system as a whole, and not structured according to its conceptualized subsystems, therefore we have merged the general and parallel MHPSS system for refugees into one “refugee MHPSS system” in Figure 1. The figure now emphasises our focus on the refugee MHPSS system and consequently aligns better with our presentation of the results. Additionally, we have used the terms of Figure 1 as headers to structure our results section (i.e., Socio-cultural, political, and economic context; Health system inputs: refugee MHPSS system; Intermediate health system outcomesgoals: responsiveness; Final goals: improve mental healthMental health outcomes) to further enhance alignment with our conceptual framework.
- For clarification our analysis was focused on HSR for refugees, and Syrian refugees in particular, and we did not compare this to citizens. Such a comparison, however, could generate useful insights and therefore we have added this as a sixth limitation of our study and recommendation for future research (see “limitations” at the end of our Discussion section).

  Thank you again for your feedback and useful comments. We hope you find our changes satisfactory.   Yours sincerely,   Aniek Woodward (and also on behalf of all co-authors)

# Reviewer report: Reviewer response for version 1

**DOI:** 10.21956/openreseurope.16535.r33397

**Published:** 2023-07-21

**Authors:** Akihiro Seita

**Affiliations:** 1 Department of Health, United Nations Relief and Works Agency, Amman, Jordan

Dear Authors,

 Thank you for providing this highly engaging and significant manuscript. While I am not an expert in MHPSS, working with refugees has given me a deep appreciation for your dedicated efforts in addressing the crucial issue of mental health among Syrian refugees. Your manuscript holds invaluable information for the global health and humanitarian communities, and it should undoubtedly be indexed.

 However, I would like to raise several key strategic issues related to your analysis and recommendations:

 Firstly, I found the grouping of the eight host countries to be a potential concern. Among these countries, we have four high-income European nations (Germany, Netherlands, Sweden, and Switzerland) with a GDP per capita ranging from USD 50,000 to 90,000, while the remaining three Middle Eastern countries (Egypt, Jordan, and Lebanon) have middle-income status, with a GDP per capita of USD 3,000 to 5,000. Grouping all eight countries together and providing common recommendations may not be practical, considering the substantial differences in the development, financing, and functionality of their respective health systems.

 This distinction is particularly crucial when it comes to intermediate health goals, encompassing seven access and coverage indicators. Access and affordability of health services vary significantly between countries with achieved universal health insurance coverage (such as the four high-income countries) and those still developing their health insurance systems (like the three middle-income countries). While the challenges in health system responsiveness you have identified might share similar principles across all eight countries, the practicality and strategic responses to these issues differ significantly.

 Hence, I believe it would be more appropriate to discuss the subject matter separately for high-income and middle-income countries. In this regard, splitting your manuscript into two manuscripts—one focusing on the four high-income countries and the other on the three (or four, including Turkey) middle-income countries—would provide a more comprehensive and targeted approach.

 Secondly, I would like to address the nature of the recommendations. I genuinely appreciate and value your categorization of recommendations into various chapters, such as socio-cultural, health system inputs, intermediate health system outcomes, and others. However, it may be necessary to reconsider and revise the way these recommendations are presented. Please find my specific comments below.

 Regarding the first recommendation to "strengthen the socio-economic situation of refugees," I wholeheartedly agree with its importance. However, this recommendation raises another issue, as outlined below.

 The second, fifth, and sixth recommendations (related to mental health workforce, national health information systems, and national funding for mental health) have significant resource implications. While these recommendations are undoubtedly critical, especially for middle-income countries with limited resources and health systems struggling to meet the increasing healthcare needs of their populations, they may not be realistic or strategic. While these recommendations may be more relevant to high-income countries with comparatively stable national health system financing, it is important to note that economic difficulties can also impact high-income countries.

 Therefore, I suggest focusing on immediate actions that resource-constrained middle-income countries can undertake by maximizing the utilization of existing resources, both in terms of finance and human resources, to improve MHPSS for refugees. This is another reason why I propose splitting the manuscript into two manuscripts—one for high-income countries and the other for middle-income countries.

 Thirdly, I would like to address the issue of access to mental health services for both citizens and refugees. If I may have missed it in your manuscript, I was unable to determine whether Syrian refugees have equal access to MHPSS compared to the host countries' citizens. Ensuring that MHPSS is inclusive and based on the right to health is of utmost importance.

 Lastly, while I deeply appreciate your manuscript as an analysis of health systems response, my experience highlights the critical role of engagement and collaboration with non-health sectors in providing MHPSS (and all healthcare) to refugees. Refugees often face social and economic marginalization, leaving them vulnerable to various risks. Therefore, I highly appreciate your first recommendation regarding the improvement of the socio-conditions for refugees. If possible, it would be beneficial to mention the engagement or collaboration with other sectors, particularly those involved in social security or social safety networks, in the provision of MHPSS care for refugees in these countries.

 Once again, I highly value your extremely important manuscript, which I believe should be indexed. I hope my comments above prove helpful to you.

Is the study design appropriate and does the work have academic merit?

Yes

Is the work clearly and accurately presented and does it cite the current literature?

Partly

If applicable, is the statistical analysis and its interpretation appropriate?

Yes

Are all the source data underlying the results available to ensure full reproducibility?

Yes

Are the conclusions drawn adequately supported by the results?

Partly

Are sufficient details of methods and analysis provided to allow replication by others?

Partly

Reviewer Expertise:

Health systems, humanitarian assistance in health

I confirm that I have read this submission and believe that I have an appropriate level of expertise to confirm that it is of an acceptable scientific standard, however I have significant reservations, as outlined above.

# Response

**Published:** 2024-05-01

**Authors:** Aniek Woodward

**Affiliations:** KIT Royal Tropical Institute, The Netherlands

Dear Dr. Seita,   Thank you for taking the time to review our paper. In response to your valuable feedback we have made the following changes to our paper:

- While we appreciate your recommendation to split our paper into two papers (one covering high-income countries and another middle-income countries), we prefer to keep all results in one paper. The reason is that we think a key value and novelty of our paper (and the STRENGTHS study at large) is the comparison of findings for Syrian refugees displaced across very different settings. Our study showed many similarities in findings across very different socio-economic country settings, which increases the transferability of results to a broad range of settings. Naturally, there were some differences between the countries due to their varying contexts, which we had highlighted in our results and discussion where necessary.
- We appreciate the point you make that most of our recommendations require significant financial input and system change, which will be difficult to achieve in the long run. However, as the focus is health system responsiveness we believe that our recommendations for system improvements are in line with this focus. Adding short-term solutions we believe would undermine our message of the need for system change.
- We fully understand your comment that “I was unable to determine whether Syrian refugees have equal access to MHPSS compared to the host countries' citizens” as we did not compare access levels between Syrian refugees and host country citizens. Such a comparison, however, could generate useful insights and therefore we have added this as a sixth limitation of our study and recommendation for future research (please see the Limitations section at the end of the Discussion).  
- Following your suggestion to mention the engagement or collaboration with other sectors in our Recommendations section, we included the need for cross-sectoral collaboration as part of our first recommendation on strengthening the socio-economic situation of refugees.

  Thank you again for your feedback and useful comments, which we feel have significantly strengthened the manuscript. We hope you find our changes satisfactory.   Yours sincerely,   Aniek Woodward (on behalf of all co-authors)
